# Measuring antenatal care timing and content across 131 low-income and middle-income countries, 1995–2023: a systematic analysis of trends

**DOI:** 10.1016/S2214-109X(26)00010-0

**Published:** 2026-04-15

**Authors:** Anna Gage, Madeleine Conrad, Megan Knight, Aduragbemi Banke-Thomas, Corinne Bintz, Kelly Bienhoff, Rakhi Dandona, M Ashworth Dirac, Thomas Glucksman, Simon I Hay, Ali H Mokdad, Bancy Ngatia, Marie-Jeanne Offosse, Bridget Stollfus, Asnake Worku, Christopher J L Murray, Enis Barış, Nicholas J Kassebaum, Annie Haakenstad

**Affiliations:** a Institute for Health Metrics and Evaluation, Hans Rosling Center for Population Health, University of Washington, Seattle, WA, USA; b Department of Epidemology, Emory University, Atlanta, GA, USA; c Health Systems Insight, Ouagadougou, Burkina Faso; d London School of Hygiene & Tropical Medicine, London, UK; e Public Health Foundation of India, Delhi, India; f The National Data Management Center for Health, Ethiopian Public Health Institute, Addis Ababa, Ethiopia; g Department of Anaesthesiology & Pain Medicine, University of Washington, Seattle, WA, USA; h Department of Global Health, University of Washington, Seattle, WA, USA; i Department of Health Metrics Sciences, University of Washington, Seattle, WA, USA; j Department of Family Medicine, University of Washington, Seattle, WA, USA

## Abstract

**Background:**

Timely and comprehensive antenatal care can improve maternal and perinatal health by enabling early detection and treatment of pregnancy complications. We estimated the content and timing of antenatal care visits for 131 low-income and middle-income countries (LMICs) from 1995 to 2023.

**Methods:**

We systematically identified population-representative data sources capturing antenatal care timing and content. We selected five widely measured indicators of antenatal care screening and prevention services: iron supplementation, measurement of weight and blood pressure, and provision of blood and urine samples. We modelled the proportion of women with a livebirth reporting all five items (antenatal care content proportion), mean number of items reported (antenatal care content mean), proportion with first trimester antenatal care attendance, and mean number of antenatal care visits using spatiotemporal Gaussian process regression.

**Findings:**

Across LMICs, the proportion of women with a livebirth who initiated antenatal care in the first trimester increased from 46·2% (95% uncertainty interval 42·2–49·9) in 1995 to 63·7% (62·8–64·5) in 2023, and the average number of antenatal care visits during pregnancy rose from 3·7 (3·4–4·0) to 6·0 (5·9–6·1). In 2023, 69·3% (68·4–70·1) of women with a livebirth received all five antenatal care items, a major increase from 1995 (25·9% [24·1–27·8]). In 2023, the proportion of women who attended antenatal care but did not receive all five items (26·2% [25·5–27·0]) exceeded those who did not attend antenatal care at all (7·1% [6·5–8·1]). The receipt of all five items also varied by geography: 79·2% (77·6–80·5) in Latin America and the Caribbean received all five items, compared with 59·8% (58·4–60·9) of women in sub-Saharan Africa.

**Interpretation:**

Despite improvements in the receipt of basic elements of antenatal care from 1995 to 2023, health systems continue to miss opportunities to deliver important care to women who attend antenatal care. The identified gaps in coverage should be targeted for improvement to support receipt of timely and effective health care that improves maternal and neonatal health.

**Funding:**

The Gates Foundation.

## Introduction

Over the past three decades, the use of antenatal care has increased markedly in low-income and middle-income countries (LMICs), creating new opportunities to improve maternal and newborn health. From 1995 to 2023, the proportion of pregnant women who attended at least four antenatal visits (ANC4) increased from 34% to 68% in LMICs.^1^ In 2016, WHO issued an updated recommendation for all pregnant women to receive a minimum of eight antenatal care contacts, replacing the previous four-visit guidance.^2^ Antenatal care serves as an entry point for continued care throughout the perinatal period, as women who attend antenatal care are more likely to deliver in health facilities and attend postnatal care.^3^ Attending antenatal care is associated with reduced neonatal mortality and probability of low birthweight, stunting, and underweight.^4^, ^5^

The effectiveness of antenatal care depends on the timing and quality of these visits. Early initiation of antenatal care during the first trimester of pregnancy is crucial for timely detection and management of pregnancy-related risks and existing chronic conditions.^6^, ^7^ Blood pressure monitoring is used to identify life-threatening pre-eclampsia.^8^ Testing and treatment for asymptomatic bacteriuria can prevent preterm birth and lower the risk of maternal and neonatal sepsis.^2^ Iron-folate supplementation during pregnancy can prevent maternal anaemia and lowers the risk of low birthweight.^9^ Monitoring weight over the course of pregnancy can identify inadequate and excessive gestational weight gain and risk factors for stillbirth, preterm birth, caesarean section, and child overweight.^10^ However, despite the measured increase in antenatal care coverage, expanded access does not necessarily guarantee the delivery of these essential screenings and interventions that are crucial to realising health gains.

**Research in context**

**Evidence before this study**

We conducted a review of global health databases and literature for existing estimates of antenatal care timing and content of care. We searched PubMed from Jan 1, 1995, to April 7, 2025 using the search terms “antenatal care”, “prenatal care”, “content of care”, “quality of care”, “initiation”, and “timing” with no language restrictions. All databases and hundreds of articles reported on binary antenatal care use (at least one visit or at least four visits). We identified nine studies that compared antenatal care content across ten or more countries, most commonly reporting on the receipt of blood pressure monitoring, urine samples, and blood samples. We found three studies reporting on the timing of antenatal care initiation across ten or more countries. Although these studies revealed global variation in the timeliness and care content of antenatal care, all these analyses had limitations. Namely, all identified studies on the content of antenatal care were limited to cross-sectional comparisons using the most recent household survey data and all were limited in geographical scope.

**Added value of this study**

Comparable estimates of antenatal care content and timing are crucial to identifying and addressing gaps in maternal health care. We used all available data from 643 surveys to estimate the receipt of five clinical activities identified by WHO as proxies of provision of recommended antenatal care. We also estimated the timing of antenatal care initiation and the mean number of antenatal care visits among pregnant women. For the first time, we systematically modelled the trends of these indicators over 1995–2023 across 131 low-income and middle-income countries, developing comparable estimates over time and across geographies.

**Implications of all the available evidence**

Although coverage and content of antenatal care have improved substantially since 1995, important gaps remain in the delivery of essential services such as iron supplementation and urine testing, even among women who attend antenatal care. Progress in antenatal care has not been uniform: some regions, such as Latin America and the Caribbean, have achieved high coverage of recommended antenatal care content, whereas others, including sub-Saharan Africa, continue to lag behind. In countries with high maternal and neonatal mortality, opportunities for early intervention are being missed because many women attend their first antenatal care visit after the first trimester. Further improvements in maternal and newborn health will depend not only on increasing antenatal care coverage but also on closing persistent gaps in the content and timeliness of care. Boosting the quality of antenatal care is thus important for improving maternal and neonatal health and serves as a potentially high-impact, cost-effective approach for donors and policy makers.

Existing literature has exposed shortcomings in the receipt of antenatal care content and delayed initiation of antenatal care. For example, a cross-sectional analysis of 91 LMICs showed that 27% of women who attended antenatal care did not have their blood pressure measured or provide blood and urine samples.^11^ Another cross-sectional analysis of 54 countries found that less than 50% of women who attended antenatal care initiated care during the first trimester.^6^ Other multi-country studies have shown gaps in the provision of iron–folate supplementation and other preventive care.^5^, ^12^, ^13^, ^14^ Although these studies provide insights into cross-country variation in antenatal care content and timing, they are limited in scope—focusing on select indicators, restricted sets of countries, and single time points—leaving major gaps in understanding broader trends over time and between regions. This information is essential to tracking how content and timing have differed as countries scaled antenatal care coverage.

Our study aims to fill these gaps by using all available data to model multiple antenatal care indicators, including content, timing, and quantity, across 131 LMICs from 1995 to 2023. With these estimates, policy makers can address gaps and target interventions to improve the timeliness and effectiveness of antenatal care for better maternal and newborn health.

## Methods

### Overview

We used 643 population-representative surveys to describe the receipt of five recommended antenatal care items, initiation of antenatal care in the first three months of pregnancy, and the mean number of antenatal care visits. We used spatiotemporal Gaussian process regression to model these indicators for 131 LMICs over 1995–2023. This study is compliant with the GATHER requirements (appendix pp 3–4). A modelling flowchart is in the appendix (p 14). Because this study only used secondary, de-identified data, ethical approval was not required.

### Data seeking

We conducted a comprehensive search of relevant data sources in the Global Health Data Exchange, a repository for population health surveys, vital registration systems, and other health data. We reviewed 2296 potential sources and included those with information on at least one of the antenatal care indicators (table) during past pregnancies from population-representative surveys, vital registration sources, and reports from 1995 to 2023. We selected 1995 as the start year given the relative paucity of data before that date and the release of WHO antenatal care recommendations in 1996.^15^ We excluded data sources that were not population-representative, only asked about current pregnancies, did not include interview date or date of birth, or were in high-income countries as per the World Bank 2025 classifications.^16^ The data that met the inclusion criteria and were retained in the analysis were all surveys.

**Table:** Indicator definitions

	**Definition**
Iron supplementation	The proportion of women with a livebirth who received iron–folic acid or iron-only tablets or syrup during pregnancy
Weight measured	The proportion of women with a livebirth who had their weight measured at least once during antenatal care
Blood pressure measured	The proportion of women with a livebirth who had their blood pressured measured at least once during antenatal care
Blood sample	The proportion of women with a livebirth who provided a blood sample at least once during antenatal care
Urine sample	The proportion of women with a livebirth who provided a urine sample at least once during antenatal care
Antenatal care content proportion	The proportion of women with a livebirth that had all of the following: iron supplementation, weight measured, blood pressure measured, blood sample, and urine sample
Antenatal care content mean	The mean number of antenatal care items (iron supplementation, weight measured, blood pressure measured, blood sample, and urine sample) received among all women with a livebirth
Early antenatal care initiation	The proportion of women with a livebirth who had their first antenatal care visit during the first trimester
Mean antenatal care visits	The mean number of antenatal care visits received during pregnancy among women with a livebirth

### Indicator selection

Selected antenatal care content indicators met three criteria for inclusion: recommended in 2016 WHO antenatal care guidelines^2^ with a history of recommendation throughout the study period,^15^ applicable to every world region, and reported in at least 200 unique data sources. Five indicators of clinical care services met these criteria: iron supplementation, weight measured, blood pressure measured, and testing of blood and urine. Two composite indicators (proportion receiving all five services [antenatal care content proportion] and mean number of services received [antenatal care content mean]) and two timing (proportion first trimester initiation and mean number of visits attended) were also constructed. We use the terms contact and visit interchangeably.

### Data extraction and processing

Many surveys, including the Demographic and Health Surveys and Multiple Indicator Cluster Surveys, ask women about antenatal care received for their most recent pregnancy within the past 3–5 years. To avoid potential cohort effects and reduce recall bias, we restricted the data to only include the most recent livebirth within 2 years of the survey. Among those respondents, iron supplementation coverage was extracted among all survey respondents who reported a livebirth; other indicators were extracted among those attending at least one antenatal care visit, as only these respondents were asked about these services.

Our final dataset included 643 sources covering 1086 location-years and 59·2 million pregnancies. Of the 643 sources, 73 were missing data on weight measurement, 61 were missing data on iron supplementation, and seven were missing other indicators. To include these abbreviated data sources in the composite indicator models, we predicted the value of the full composite from an alternate definition with a shorter index using a meta regression–Bayesian, regularised, trimmed crosswalk approach. Details are available for number of data sources by indicator and geography (appendix pp 5–9) and for crosswalk development and implementation (appendix pp 9–13).

### Modelling

We separately modelled each indicator (table) for 1995–2023 using spatiotemporal Gaussian process regression (ST-GPR), a three-stage statistical approach designed to produce consistent global estimates with robust uncertainty. First, we fit a linear mixed effects regression model with covariates ANC4 coverage, health worker density, and Healthcare Access and Quality Index, predicting a set of initial estimates based on the association with these covariates. Second, locally weighted polynomial regression smoothed the residuals between the data and the first-stage model predictions over geography and time, borrowing strength from data in nearby locations and years to improve estimates when data were sparse. Third, Gaussian process regression further smoothed estimates and quantified uncertainty by incorporating the variance in our input data and the differences between the first and second stage estimates. This approach assumes temporal and geographical patterns are informative for estimating missing values and covariate relationships hold across settings. Further details on ST-GPR and covariates are included in the appendix (pp 14–16).

After modelling, we ensured that the antenatal care content proportion did not exceed the lowest estimated value among the five component indicators for a given location and year, maintaining logical consistency as described in the appendix (p 15). We then multiplied the indicator estimates by the proportion of women who received at least one antenatal care visit to estimate the receipt of the indicators among all women with a livebirth. Region aggregates were weighted by livebirths per country.^17^

We report levels and time trends for the content indicators, composite indicators, and timing indicators for 131 LMICs from 1995 to 2023. Antenatal care attendance proportion is also presented for comparison; its methods are described in the appendix (p 17). All indicators are presented among all women of any age with a livebirth.

### Uncertainty

We sampled 1000 random draws from the posterior distribution of the Gaussian process regression for each location and year. All adjustment and aggregation steps were conducted at the draw level; uncertainty intervals were calculated by taking the 2·5th and 97·5th percentile values across the draws.

### Secondary analyses

We sought to understand whether the antenatal care content and timing indicators were more correlated with health outcomes than was ANC4, historically the most widely estimated and reported measure of antenatal care. We selected three outcomes from the Global Burden of Diseases, Injuries, and Risk Factors Study database: maternal mortality ratio (MMR), neonatal mortality (NMR), and stillbirth rate (SBR). We primarily focused on comparing the correlation between ANC4 and the antenatal care content mean. Additional analysis of the associations with the health outcomes is included in the appendix (pp 17–19).

### Role of the funding source

The funder of the study had no role in study design, data collection, data analysis, data interpretation, or writing of the report.

## Results

**Figure 1:**
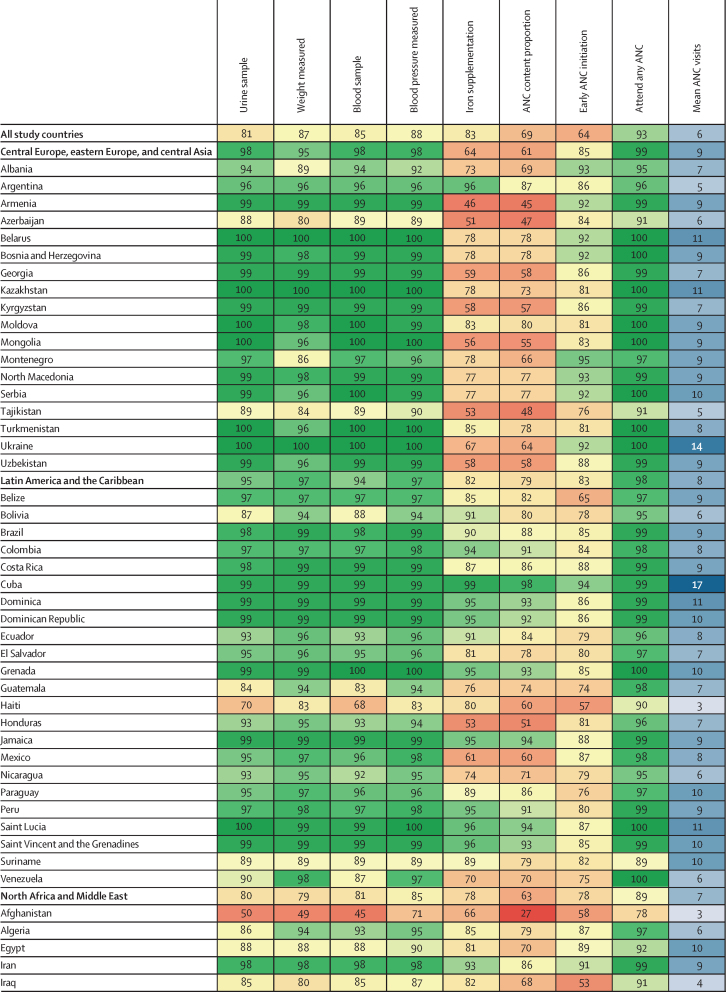

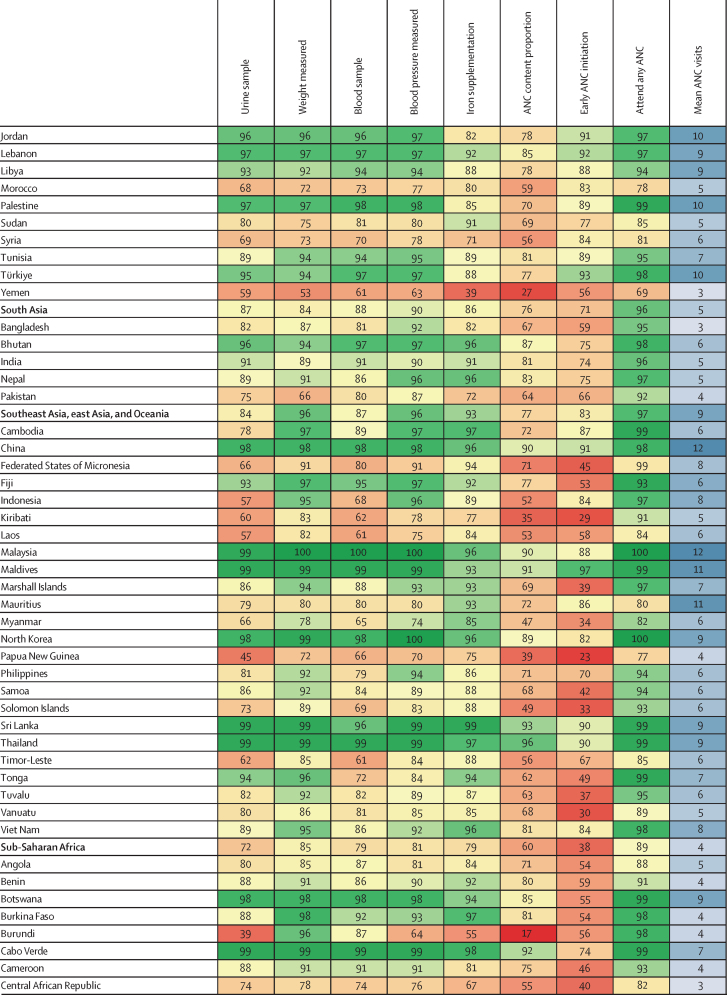

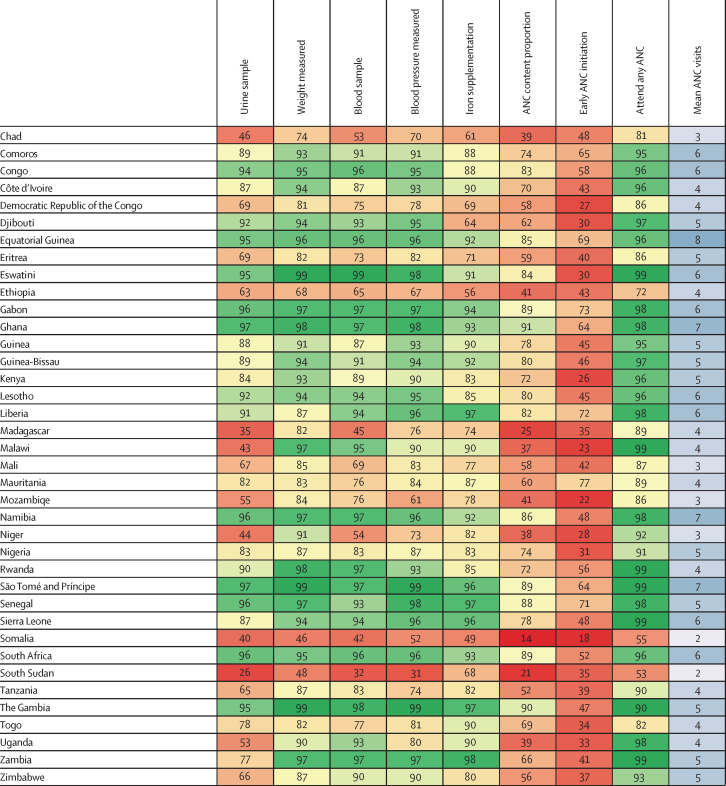
ANC content and timing by location, 2023 ANC=antenatal care.

Antenatal care indicator estimates for all LMICs in 2023 are shown (figure 1). In 2023, 69·3% (95% uncertainty interval [UI] 68·4–70·1) of women with a livebirth across 131 LMICs received all five basic antenatal care content indicators at least once: blood pressure measurement, blood sample, urine sample, weight measurement, and iron supplementation. Among women attending any antenatal care, this figure was 73·8% (72·9–74·5; appendix pp 36–38). In 2023, 63·7% (62·8–64·5) of women with a livebirth initiated antenatal care in the first trimester and attended an average of 6·0 (5·9–6·1) antenatal care visits throughout pregnancy.

**Figure 2:**
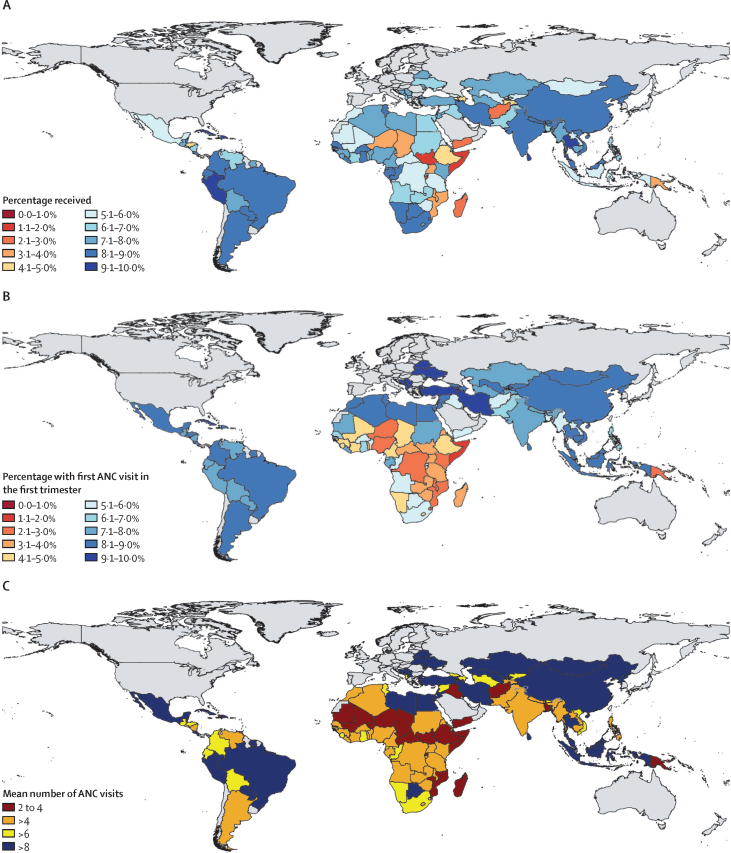
Antenatal care content and timing, 2023 Share of livebirths in which all elements of ANC content were reported (A), share of livebirths in which the first ANC visit occurred in the first trimester (B), and average number of ANC visits (C). ANC=antenatal care.

Maps of 2023 indicator estimates show substantial variation in antenatal care timing and content across LMICs in 2023 (figure 2). Initiation of antenatal care was frequently delayed after the first trimester in Papua New Guinea and many sub-Saharan African countries, although several west and central African countries (eg, Liberia and Gabon) had relatively higher (compared with the LMIC average) early initiation. In several eastern European countries, over 90% of women attended antenatal care in their first trimester. In 114 of 131 countries studied, average antenatal care visits during pregnancy exceeded the historically recommended four visits; in 41 of these 114 countries, the mean exceeded the updated recommendation of eight visits. Many of the countries below four mean visits are in the Sahel region of sub-Saharan Africa. Although nearly all pregnant women in Cuba (98·0% [97·4–98·5]) received all five indicators, fewer than 20% of pregnant women in Somalia and Burundi received all five.

**Figure 3:**
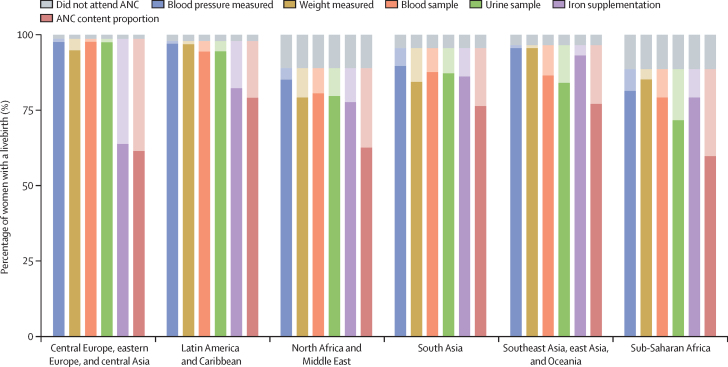
Antenatal care content and timing by region, 2023 Lighter shades show the difference between the percentage receiving the content item and the percentage not using ANC, thus representing the percentage with missed opportunities to receive the item. ANC=antenatal care.

Individual antenatal care content indicators by region in 2023 are presented (figure 3). Across indicators, blood pressure (88·2% [95% UI 87·2–88·9]) and weight measurement (87·5% [86·3–88·5]) were the most commonly received services reported by women with a livebirth. Urine sample test was the least common, with 81·4% (80·5–82·1) of women providing a urine sample during their pregnancy, although this differed substantially across regions. In 2023, 97·6% (96·7–98·2) of women in LMICs across central Europe, eastern Europe, and central Asia provided a urine sample during pregnancy, compared with only 71·7% (70·0–73·1) of women in sub-Saharan Africa. There were large gaps in iron supplementation in central Europe, eastern Europe, and central Asia, where only 63·9% (60·9–66·7) of women received supplements. Across LMICs, the proportion of women who attended antenatal care but did not receive all five items (26·2% [25·5–27·0]) exceeded the proportion of women who did not attend antenatal care during pregnancy (7·1% [6·5–8·1]); this pattern was observed across each region as well as overall. Gaps between antenatal care attendance and antenatal care content proportion were large particularly in countries in east and central Africa and Oceania (appendix p 34).

**Figure 4:**
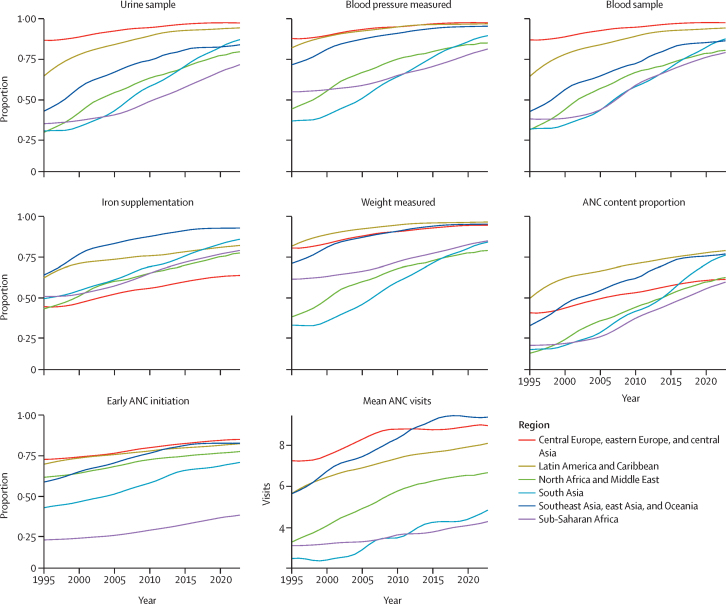
Trends in antenatal care content and timing by region, 1995–2023 ANC=antenatal care.

From 1995 to 2023, the proportion of women reporting each antenatal care item increased across all regions, and regional disparities narrowed for every indicator (figure 4). Of the five content indicators, blood sample had the largest increase, rising from 40·5% (95% UI 36·5–44·3) in 1995 to 84·7% (83·7–85·4) in 2023. The proportion of women receiving all five items increased the most in south Asia, from 17·9% (16·1– 19·8) in 1995 to 76·4% (74·4–77·8) in 2023. Although early antenatal care initiation remains low in sub-Saharan Africa, it improved by 15·4 percentage points (12·9–17·8) between 1995 and 2023. In 2016, when WHO updated their recommendation from four to eight antenatal care contacts, 41 countries already exceeded eight average visits, whereas two countries, Viet Nam and Colombia, had fewer than eight on average but met that target by 2023. Changes between 1995 and 2023 by country are shown in the appendix (pp 26–32): Peru had the largest absolute improvement in the antenatal care content proportion, from 9·5% (8·0–11·1) to 91·2% (89·2–92·9), whereas Rwanda improved the most in relative terms, from 0·9% (0·7–1·1) to 72·1% (67·9–75·7).

**Figure 5:**
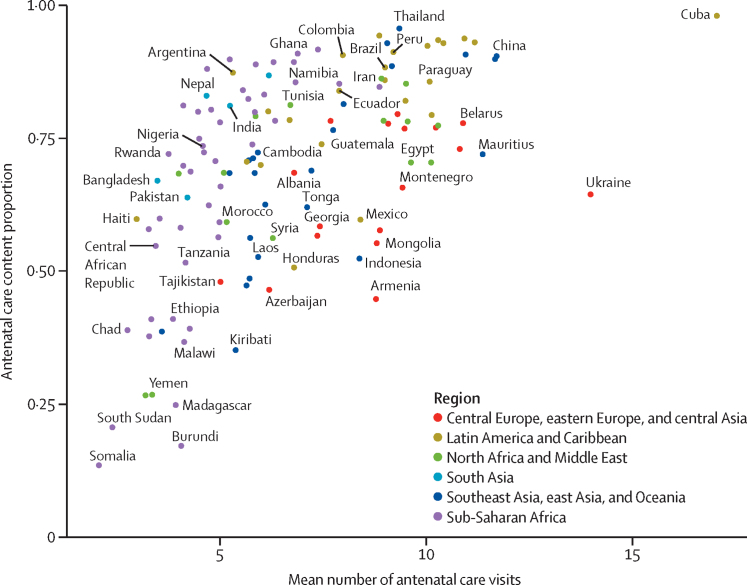
Receipt of all antenatal care items (antenatal care content proportion) versus mean number of antenatal care visits, 2023

Antenatal care visits by the content proportion showed that countries with a higher number of average visits tended to have higher proportions of women receiving all five items in 2023 (figure 5). However, notable disparities in content persisted among countries with a similar number of average visits. For example, women in both Thailand and Indonesia had just over eight visits on average in 2023, but just 52·4% (95% UI 46·8–57·9) of women in Indonesia received all five items, compared with 95·7% (94·5–96·6) in Thailand. In 53 countries with over four average visits and 11 countries with over eight, less than 75% of women received all five items. Countries with earlier antenatal care initiation also had higher antenatal care content proportions on average (appendix p 33).

The antenatal care content mean was more correlated with each health outcome than ANC4: –0·61 (95% UI –0·62 to –0·58) versus –0·48 (–0·51 to –0·46) for MMR; –0·84 (–0·85 to –0·83) versus –0·77 (–0·78 to –0·76) for NMR and –0·69 (–0·70 to –0·67) versus –0·64 (–0·65 to –0·62) for SBR. Additional results on the associations with health outcomes are in the appendix (pp 34–36).

## Discussion

Over the past 30 years, as coverage of antenatal care has increased in LMICs, so too has receipt of basic elements of care crucial to healthy pregnancies. Across world regions, large disparities between LMICs in antenatal care content narrowed between 1995 and 2023, with south Asia in particular making large strides in closing the gap with regions with higher social and economic development. Despite this great progress, many women in 2023 were still not receiving basic elements of care during pregnancy in LMICs, even if they attended antenatal care. We identified gaps in weight measurement across multiple regions, low iron supplementation in eastern Europe and central Asia, and delayed initiation of antenatal care in sub-Saharan Africa. Together, these gaps present missed opportunities to provide care that could avert preventable newborn and maternal morbidity, mortality, and stillbirths.

One of our most notable findings is that in many LMIC settings, missed opportunities during antenatal care visits accounted for a larger share of gaps in content coverage than non-attendance, consistent with other studies.^11^, ^12^ We found that in 53 countries where women attended antenatal care on average more than four times, less than 75% of women received all five basic items. This finding suggests that health systems are failing to reliably deliver a minimal package of care, despite women physically presenting for services. Our finding that the mean proportion of items received is more correlated with health outcomes than ANC4 coverage further shows that antenatal care content is essential to translating contact into health gains.

Delayed initiation of antenatal care after the first trimester remains common, particularly in countries with high maternal and neonatal mortality, a finding consistent with other studies.^6^, ^14^ Early initiation is critical for education, nutritional assessments, and management of pre-existing conditions such as hypertension, anaemia, diabetes, and infections.^14^ Early initiation can connect women to broader maternal health interventions, which can be key to preventing indirect maternal deaths.^18^ Policies in countries with widespread early initiation such as Türkiye and Cuba could provide useful examples for connecting women to care.

Despite global progress, antenatal care coverage gaps persist in some areas: in 17 countries, the mean number of visits was below the historically recommended four visits in 2023, and in 73 more countries, the mean number of visits was below the updated recommendation of eight. Low utilisation might reflect barriers to access, such as cost, distance, or social or cultural norms, but it could also reflect a rational response by women to low-value services: if care is perceived as ineffective, women might delay or avoid seeking antenatal care altogether.^19^ Coverage was low in countries with recent conflict or instability, including Somalia, South Sudan, and Haiti, highlighting the crucial role of security in supporting care availability and use.^20^

Substandard antenatal care can stem from supply shortages, staffing gaps, and poor adherence to guidelines. Barriers to iron supplementation uptake, for example, include weak supply chains, perceived lack of value, and concerns about side-effects such as nausea.^21^ Gaps in the provision of blood and urine samples can reflect poor access to laboratories, reagents stock-outs, and broken equipment.^22^ Out-of-pocket costs could also deter receipt if there are additional fees for conducting laboratory tests or purchasing iron supplements. Measurement of weight and blood pressure does not require as many supplies as the other three content indicators; strategies to improve these could focus more on patient communication and adherence to guidelines. In many LMICs, providers often work in resource-constrained environments with limited support and supervision, which can further affect the consistency and quality of antenatal care delivery. Multi-pronged efforts that tackle antenatal care deficiencies across multiple dimensions of health system delivery are the most likely to address structural barriers to improve quality.

The selected indicators represent important aspects of antenatal care, but they are only a fraction of the recommended care. For example, we reported blood pressure measurement at least once, whereas WHO recommends blood pressure measurements at every visit.^2^ Other studies have found that content of care varies by the visit number and trimester of pregnancy, even for items that should occur every visit.^23^, ^24^ Our data sources also reported only on whether women provided blood and urine samples; whether these samples were appropriately analysed is unknown. Although we focus on testing and preventive care, the detection of complications in pregnancy alone is not sufficient for improving maternal and newborn health. Counselling is also a crucial component of high-quality antenatal care that directly influences health outcomes and women's engagement with care, but this was not included in our analysis. Our content estimates thus represent a signal of comprehensive antenatal care quality in LMICs; even in countries with high coverage, further improvements are probably needed as additional gaps likely remain beyond our indicators.

The indicators estimated in this study do provide important implications for policy and research. First, the identified gaps in coverage in particular countries should be targeted for improvement, especially as efficiency and resource allocation become increasingly important. Increasing the number of antenatal care visits without improving what happens during those visits risks wasting scarce resources for both women and health systems. Countries with high antenatal care content proportions, such as Cuba, Thailand, and Ghana, warrant examination to identify context-specific strategies and policies that have enabled success in low-resource settings in different regions. For example, community identification and registration of pregnant women into antenatal care in Ghana aided in early antenatal care initiation.^25^ Second, innovators interested in expanding the suite of testing and monitoring devices used during pregnancy should consider both how much testing currently occurs and whether basic testing should be the focus rather than expansion of monitoring to new areas of maternal health. Finally, as global health measurement frameworks evolve beyond 2030, it is essential that metrics of antenatal care quality are integrated into successor frameworks to the Sustainable Development Goals and ongoing initiatives such as Every Woman Every Newborn Everywhere.^26^ As the Demographic and Health Surveys were a key data source for this work, the global community must sustain their funding and strengthen other measurement systems such as health management information systems.^27^

Our analysis is not without limitations. First, all data were self-reported, so there is the potential for recall bias or social desirability bias. Although we limited the time period to 2 years to address recall bias, health record validation would strengthen the analysis. Second, we were unable to assess other effective care elements during antenatal care, including counselling, ultrasounds, folic acid supplementation, and other clinical activities. Third, the data are typically reported only among livebirths, potentially missing differences in antenatal care content among pregnancies ending in stillbirth, which is itself an important indicator of care quality. To better measure this important group, surveys should include questions on antenatal care content irrespective of the birth outcome. Fourth, our work does not assess variation in content or timing within countries, although several studies have documented inequalities with respect to wealth, parity, maternal education, and location.^6^, ^11^, ^13^ Fifth, although our data were extensive, we lacked data sources from 17 LMICs, primarily small island nations, and some sources were missing indicators for the composite, necessitating cross-walking, which could introduce bias.

Although antenatal care coverage has expanded substantially across 131 LMICs over the past three decades, persistent gaps in the content and timing of care remain. Missed opportunities during antenatal care visits often outweigh gaps caused by non-attendance, with many women attending multiple visits yet failing to receive all basic elements of care. Addressing the identified gaps is crucial for improving maternal and newborn health outcomes globally.

### Contributors

### Data sharing

Input data sources and code are available at https://github.com/ihmeuw/anc_content_timing. Estimates produced in these analyses will be available from the Global Health Data Exchange website pending publication.

## Declaration of interests

Funding for the present manuscript was provided by the Gates Foundation. AG, AH, NJK, KB, and RD have received funding from the Gates Foundation. NJK has received consulting fees from the following organisations: Bristol Meyers Squibb, Fujifilm Sonosite, and Philips Medical. RD has received funding from the Mariwala Health Initiative and USAID, reports payment as the Co-chair of the RIGHT call 8 funding committee, and has the following leadership roles: vice chair of International Stillbirth Alliance, chair of Independent Programme Oversight Committee for COPE-BP, member of the Global NIHR Global Health Research Groups Call 5 funding committee, and member of the Health Metrics Sciences MS Admissions committee at the University of Washington. All other authors declare no competing interests.

## References

[1] Global Burden of Disease Study 2023 (GBD 2023) data resources. https://ghdx.healthdata.org/gbd-2023.

[2] WHO WHO recommendations on antenatal care for a positive pregnancy experience. 2016. http://www.who.int/reproductivehealth/publications/maternal_perinatal_health/anc-positive-pregnancy-experience/en/.

[3] Geremew AB, Boke MM, Yismaw AE (2020). The effect of antenatal care service utilization on postnatal care service utilization: a systematic review and meta-analysis study. J Pregnancy.

[4] Kuhnt J, Vollmer S (2017). Antenatal care services and its implications for vital and health outcomes of children: evidence from 193 surveys in 69 low-income and middle-income countries. BMJ Open.

[5] Neupane S, Doku DT (2019). Association of the quality of antenatal care with neonatal mortality: meta-analysis of individual participant data from 60 low- and middle-income countries. Int Health.

[6] Jiwani SS, Amouzou-Aguirre A, Carvajal L (2020). Timing and number of antenatal care contacts in low and middle-income countries: analysis in the Countdown to 2030 priority countries. J Glob Health.

[7] Saad-Haddad G, DeJong J, Terreri N (2016). Patterns and determinants of antenatal care utilization: analysis of national survey data in seven countdown countries. J Glob Health.

[8] Rawlins B, Plotkin M, Rakotovao JP (2018). Screening and management of pre-eclampsia and eclampsia in antenatal and labor and delivery services: findings from cross-sectional observation studies in six sub-Saharan African countries. BMC Pregnancy Childbirth.

[9] Imdad A, Bhutta ZA (2012). Routine iron/folate supplementation during pregnancy: effect on maternal anaemia and birth outcomes. Paediatr Perinat Epidemiol.

[10] Darling AM, Wang D, Perumal N (2023). Risk factors for inadequate and excessive gestational weight gain in 25 low- and middle-income countries: an individual-level participant meta-analysis. PLoS Med.

[11] Arsenault C, Jordan K, Lee D (2018). Equity in antenatal care quality: an analysis of 91 national household surveys. Lancet Glob Health.

[12] Carvajal-Aguirre L, Amouzou A, Mehra V, Ziqi M, Zaka N, Newby H (2017). Gap between contact and content in maternal and newborn care: an analysis of data from 20 countries in sub-Saharan Africa. J Glob Health.

[13] Arroyave L, Saad GE, Victora CG, Barros AJD (2021). Inequalities in antenatal care coverage and quality: an analysis from 63 low and middle-income countries using the ANCq content-qualified coverage indicator. Int J Equity Health.

[14] Moller A-B, Petzold M, Chou D, Say L (2017). Early antenatal care visit: a systematic analysis of regional and global levels and trends of coverage from 1990 to 2013. Lancet Glob Health.

[15] WHO Mother–baby package. Implementing safe motherhood in countries. https://www.who.int/publications/i/item/WHO-FHE-MSM-94·11-Rev.1.

[16] Metreau E, Young KE, Eapen SG World Bank country classifications by income level for 2024–2025. 2024. https://blogs.worldbank.org/en/opendata/world-bank-country-classifications-by-income-level-for-2024-2025.

[17] Schumacher AE, Zheng P, Barber RM (2025). Global age-sex-specific all-cause mortality and life expectancy estimates for 204 countries and territories and 660 subnational locations, 1950–2023: a demographic analysis for the Global Burden of Disease Study 2023. Lancet.

[18] Haider MM, Siddique AB, Jabeen S (2023). Levels, trends, causes, place and time of, care-seeking for, and barriers in preventing indirect maternal deaths in Bangladesh: an analysis of national-level household surveys. J Glob Health.

[19] Pell C, Meñaca A, Were F (2013). Factors affecting antenatal care attendance: results from qualitative studies in Ghana, Kenya and Malawi. PLoS One.

[20] Amberg F, Chansa C, Niangaly H, Sankoh O, De Allegri M (2023). Examining the relationship between armed conflict and coverage of maternal and child health services in 35 countries in sub-Saharan Africa: a geospatial analysis. Lancet Glob Health.

[21] Siekmans K, Roche M, Kung'u JK, Desrochers RE, De-Regil LM (2018). Barriers and enablers for iron folic acid (IFA) supplementation in pregnant women. Matern Child Nutr.

[22] Koster W, Ondoa P, Sarr AM (2016). Barriers to uptake of antenatal maternal screening tests in Senegal. SSM Popul Health.

[23] Dandona R, Kumar GA, Majumder M, Akbar M, Prasad Dora SS, Dandona L (2023). Poor coverage of quality-adjusted antenatal care services: a population-level assessment by visit and source of antenatal care services in Bihar state of India. Lancet Reg Health Southeast Asia.

[24] Dandona R, Majumder M, Akbar M (2022). Assessment of quality of antenatal care services in public sector facilities in India. BMJ Open.

[25] Haruna U, Dandeebo G, Galaa SZ (2019). Improving access and utilization of maternal healthcare services through focused antenatal care in rural ghana: a qualitative study. Adv Public Health.

[26] Moller A-B, Patten J, Hanson C, Essén B, Jacobsson B (2026). Five decades of advancing global maternal and newborn health and rights: milestones and initiatives. In J Gynecol Obstet.

[27] Khaki JJ, Molenaar J, Karki S (2025). When health data go dark: the importance of the DHS Program and imagining its future. BMC Med.

