# Correction to *Lancet Glob Health* 2026; 14: e702-13

**DOI:** 10.1016/j.langlo.2026.103962

**Published:** 2026-08-28

*Gage A, Conrad M, Knight M, et al. Measuring antenatal care timing and content across 131 low-income and middle-income countries, 1995–2023: a systematic analysis of trends.* Lancet *2026;* ***14:*** *e702–13*—In figure 2 of this Article, the ranges provided in the keys for panels A and B were incorrect and have been updated. This correction has been made to the online version as of Aug 12, 2026.

