# Supplementary material for: Measuring antenatal care timing and content across 131 low-income and middle-income countries, 1995–2023: a systematic analysis of trends
Source: Lancet Glob Health. 2026 Apr 15;14(5):e702–13. doi: 10.1016/S2214-109X(26)00010-0 (PMC13106046; doi:10.1016/S2214-109X(26)00010-0)
Supplement: Supplementary appendix [file mmc1.pdf]

THE LANCET
Global Health

Supplementary appendix

This appendix formed part of the original submission and has been peer reviewed. We post it as supplied by the authors.

Supplement to: Gage A, Conrad M, Knight M, et al. Measuring antenatal care timing and content across 131 low-income and middle-income countries, 1995–2023: a systematic analysis of trends. *Lancet Glob Health* 2026; **14:** e702–13.

Supplementary Material: Measuring antenatal care timing and content: a systematic analysis of trends from 1995 to 2023 across 131 low-income and middle-income countries

Table of Contents

Supplementary Material: Measuring antenatal care timing and content: a systematic analysis of trends from 1995 to 2023 across 131 low-income and middle-income countries 1

Preamble...2

GATHER Checklist...3

Part 1. Supplementary methods...5

Data sources... 5
Crosswalk development and implementation.. 9
Modelling approach.. 14
Any Antenatal Care Attendance Estimation.. 17
Analysis... 17
Location codes... 20

Part 2. Supplementary results..24

Maps of individual ANC content indicators, 2023... 24
Tables presenting change over time in ANC content and timing indicators, 1995-2023........ 26
Receipt of all interventions versus early initiation 33
Difference between proportion that attended any ANC and ANC content proportion among women with a live birth, 2023... 34
Antenatal content and timing and health outcomes analysis.................................... 34
Antenatal content and timing among women who attended any ANC by country, 2023 36

References 39

Preamble

This supplementary material provides methodological detail and additional results for the antenatal content and timing indicators. The material is structured into broad sections that mimic those in the main paper. This study complies with Guidelines for Accurate and Transparent Health Estimates Reporting (GATHER) recommendations and is meant to maximize transparency of our data inputs, data processing and modelling.

GATHER Checklist

Supplementary Table 1. GATHER Checklist[1]

#	Checklist item	Description of compliance	Reference
**Objectives and funding**			
**1**	Define the indicator(s), populations (including age, sex, and geographic entities), and time period(s) for which estimates were made.	Description of indicators, definitions, relevant time periods, and populations in paper and appendix.	Main text methods and Table 1
**2**	List the funding sources for the work.	Funding sources listed in paper	Abstract
**Data Inputs**			
For all data inputs from multiple sources that are synthesized as part of the study:			
**3**	Describe how the data were identified and how the data were accessed.	Narrative description of data seeking methodology provided	Main text methods
**4**	Specify the inclusion and exclusion criteria. Identify all ad-hoc exclusions.	Inclusion and exclusion criteria provided	Main text methods
**5**	Provide information on all included data sources and their main characteristics. For each data source used, report reference information or contact name/institution, population represented, data collection method, year(s) of data collection, sex and age range, diagnostic criteria or measurement method, and sample size, as relevant.	An online list of data sources	Available from https://github.com/ihmeuw/anc_content_timing
**6**	Identify and describe any categories of input data that have potentially important biases (e.g., based on characteristics listed in item 5).	Summary of known biases included in paper	Main text discussion
For data inputs that contribute to the analysis but were not synthesized as part of the study:			
**7**	Describe and give sources for any other data inputs.	Other data inputs summarized in text and tables	Supplementary methods
For all data inputs:			
**8**	Provide all data inputs in a file format from which data can be efficiently extracted (e.g., a spreadsheet rather than a PDF), including all relevant meta-data listed in item 5. For any data inputs that cannot be shared because of ethical or legal reasons, such as third-party ownership, provide a contact name or the name of the institution that retains the right to the data.	An online list of data sources	Available at https://github.com/ihmeuw/anc_content_timing
**Data analysis**			

**9**	Provide a conceptual overview of the data analysis method. A diagram may be helpful.	Diagram provided	Supplementary Figure 14
**10**	Provide a detailed description of all steps of the analysis, including mathematical formulae. This description should cover, as relevant, data cleaning, data pre-processing, data adjustments and weighting of data sources, and mathematical or statistical model(s).	Provided in the methodological write-ups	Main text methods and supplementary methods
**11**	Describe how candidate models were evaluated and how the final model(s) were selected.	Provided in the methodological write-ups	Supplementary methods
**12**	Provide the results of an evaluation of model performance, if done, as well as the results of any relevant sensitivity analysis.	Provided in the methodological write-ups	Supplementary methods
**13**	Describe methods for calculating uncertainty of the estimates. State which sources of uncertainty were, and were not, accounted for in the uncertainty analysis.	Provided in the methodological write-ups	Main text methods
**14**	State how analytic or statistical source code used to generate estimates can be accessed.	Access statement provided	Available from https://github.com/ihmeuw/anc_content_timing.
**Results and Discussion**			
**15**	Provide published estimates in a file format from which data can be efficiently extracted.	Results are available through the Global Health Data exchange	Available at http://ghdx.healthdata.org/gbd-2023
**16**	Report a quantitative measure of the uncertainty of the estimates (e.g. uncertainty intervals).	Uncertainty intervals are provided with all results	Main text results, supplementary table 9
**17**	Interpret results in light of existing evidence. If updating a previous set of estimates, describe the reasons for changes in estimates.		Main text discussion
**18**	Discuss limitations of the estimates. Include a discussion of any modelling assumptions or data limitations that affect interpretation of the estimates.	Discussion of limitations provided in the main text	Main text methods and discussion

Part 1. Supplementary methods

Data sources

Supplementary Figure 1. Map of input data of mean ANC visits

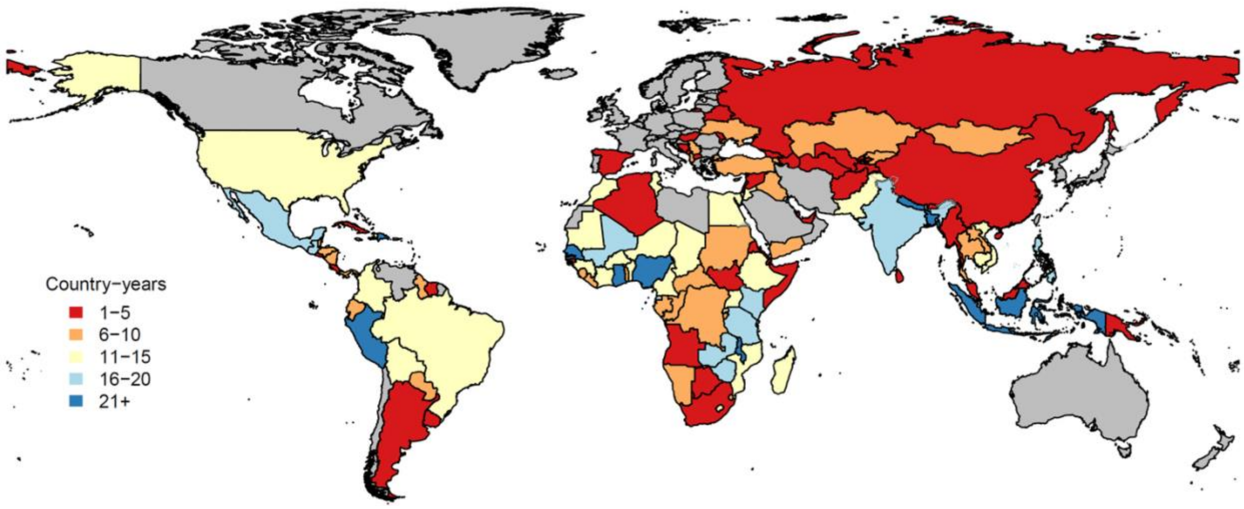

Supplementary Figure 2. Map of input data of early ANC initiation

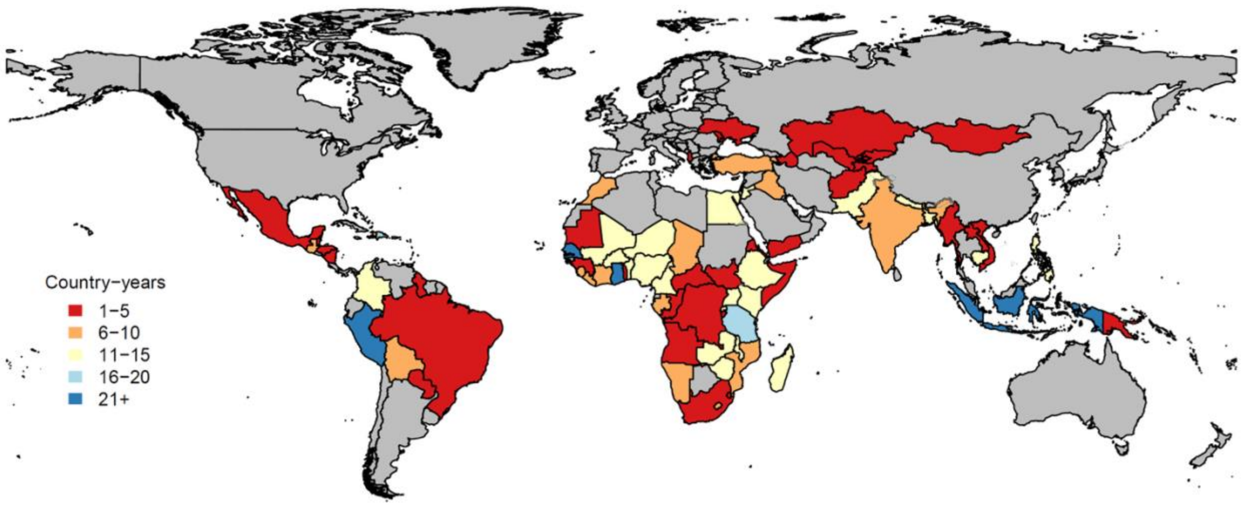

Supplementary Figure 3. Map of input data of blood pressure measurement during ANC

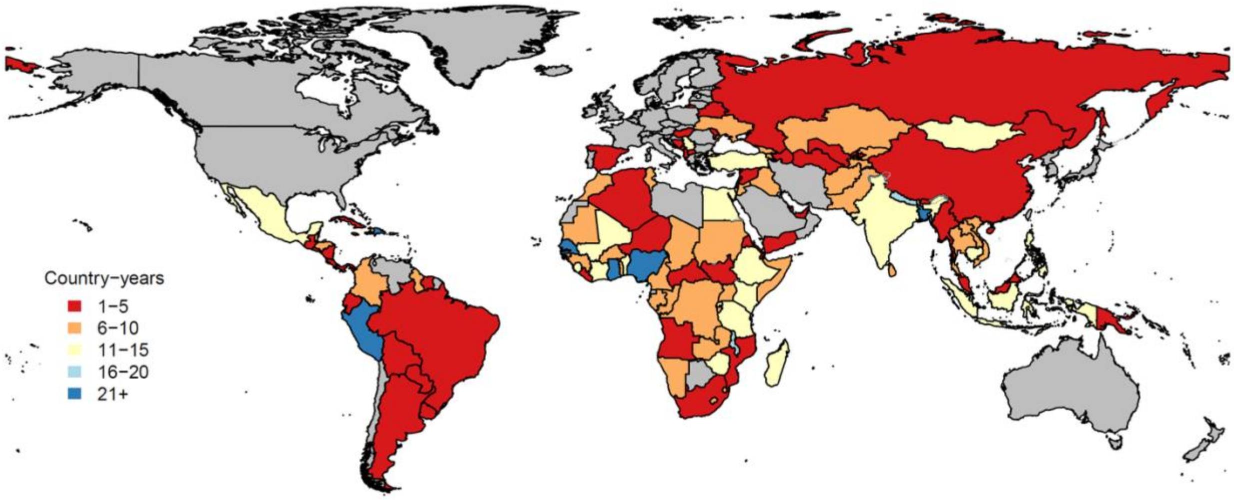

Supplementary Figure 4. Map of input data of blood sample provided during ANC

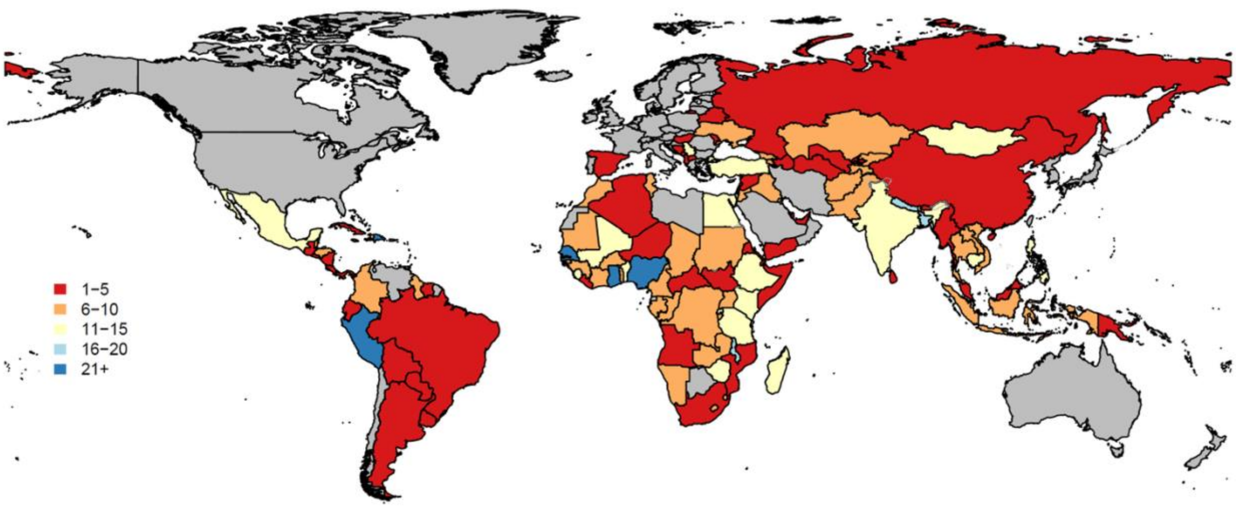

Supplementary Figure 5. Map of input data of urine sample provided during ANC

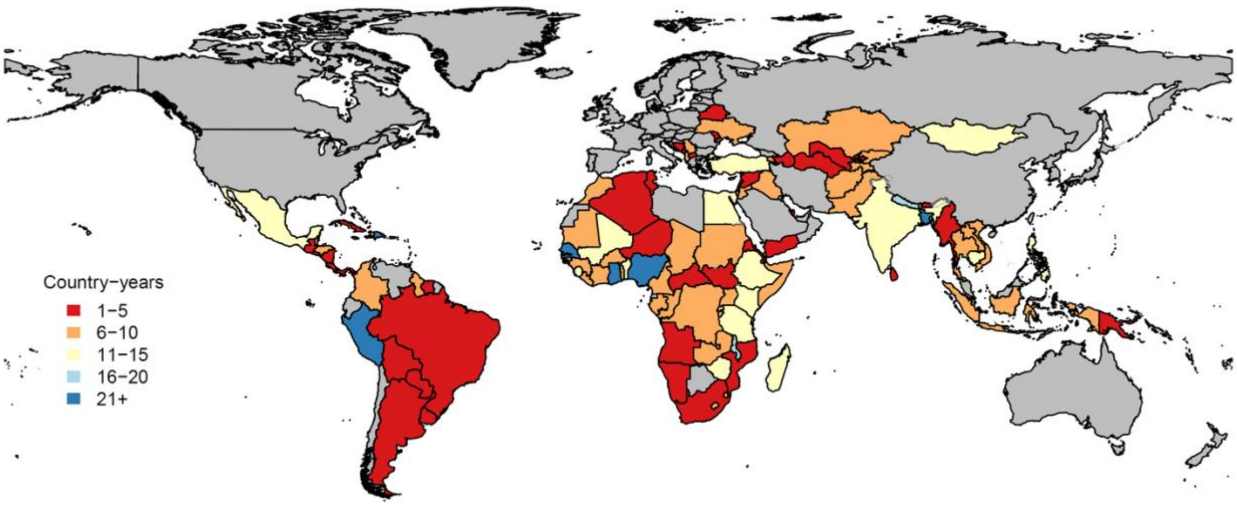

Supplementary Figure 6. Map of input data of weight measured during ANC

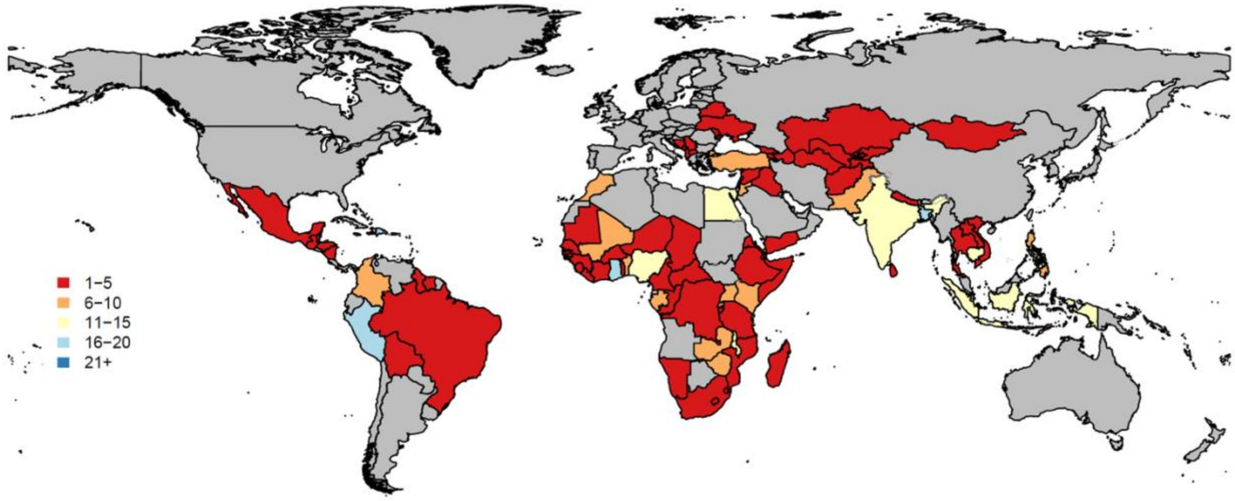

Supplementary Figure 7. Map of input data of iron supplementation

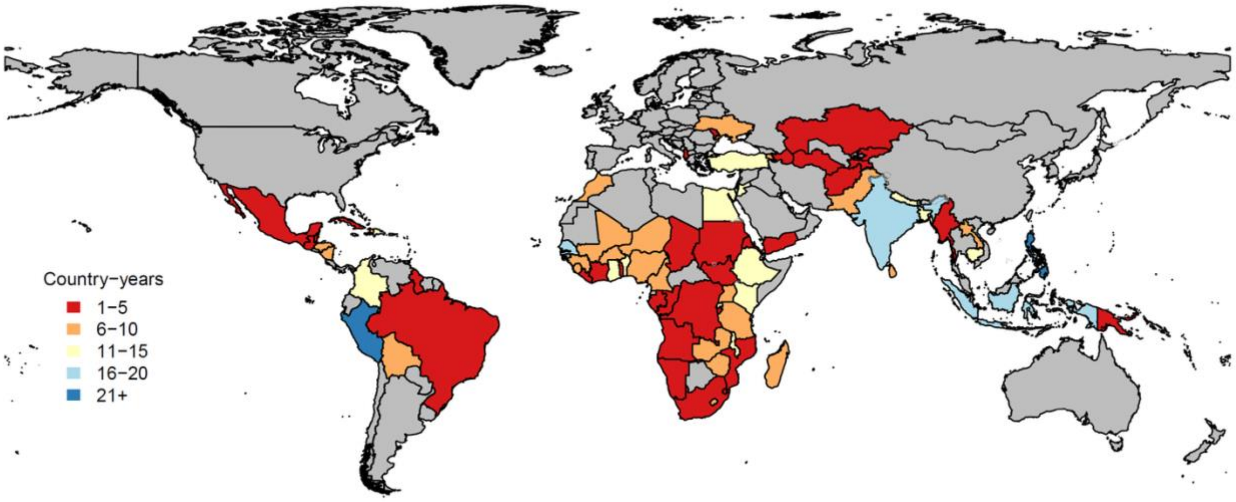

Supplementary Figure 8. Map of input data of ANC content proportion and ANC content mean

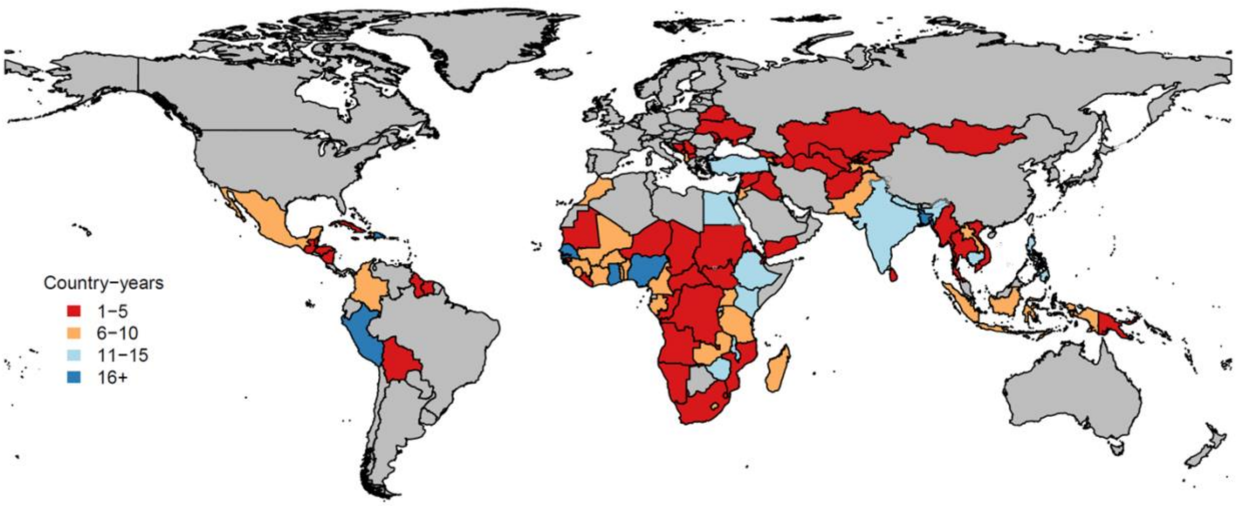

Supplementary Figure 9. Map of most recent input data source by country

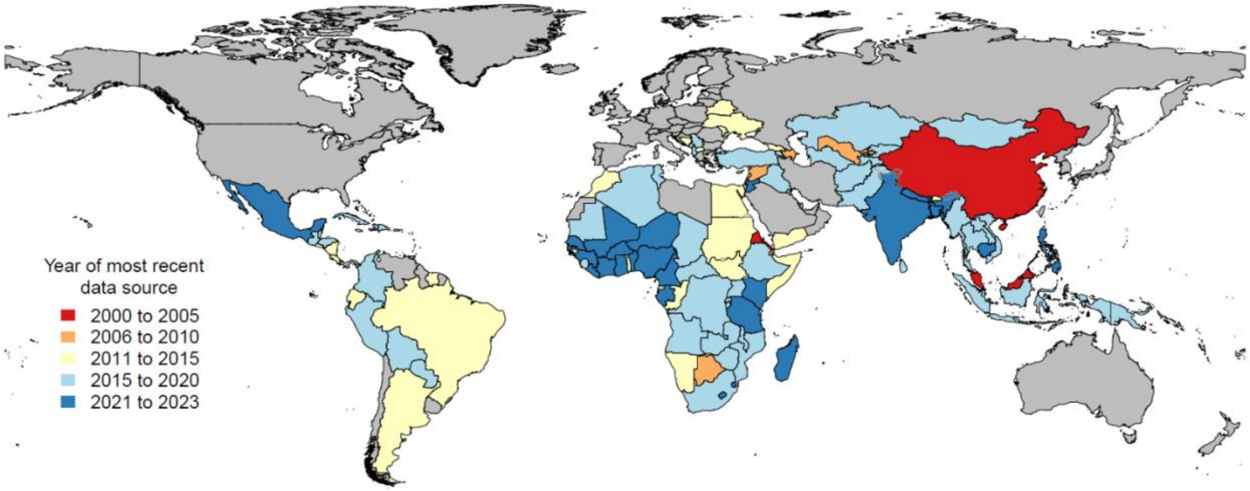

Crosswalk development and implementation

Among our 643 data sources, 73, 61, and seven were missing weight measured, iron supplementation, or other indicators, respectively. We wanted to leverage as many surveys as possible in the composite indicator models, including those which did not report all five indicators. We implemented a crosswalk approach using Meta Regression – Bayesian, Regularised, Trimmed (MR-BRT) tool from the Global Burden of Diseases, Injuries, and Risk Factors Study (GBD) in order to predict the value of the full composite index based on the information that was available in the abbreviated surveys.

First, we identified which indicators were available from the abbreviated surveys. In order to have confidence in our predictions, we only used surveys that included at least four of the five indicators; sources with fewer indicators were excluded from the prediction and modelling. We then created reference definitions using all five indicators in surveys with the data available, and shorter versions of the index, called alternate definitions in all surveys. The alternate definitions and number of data points are included in supplementary tables 1 and 2.

Using the surveys where we had both the reference and alternate definitions, we then created matched pairs between the reference and alternate definitions. We transformed the observations into logit space using the delta method, then calculated the difference between the reference definition and alternate definition and the standard error of the difference. We then used these differences in a Meta-Regression-Bayesian, Regularized, Trimmed (MR-BRT)[2] model to estimate correction factors, and finally applied these correction factors to the alternate definitions to be used in subsequent modeling steps. The matched pairs and comparisons of the original alternate definitions to the corrected values are shown in Supplementary Figures 10a -13.

In the below table of alternate definitions, the letters correspond to the interventions below, such that anc_bin_acde indicates that the data source reported on iron supplementation, blood pressure measured, blood sample, and urine sample, but did not report on weight measured.

a	Iron supplementation
b	Weight measured
c	Blood pressure measured
d	Blood sample
e	Urine sample

Supplementary Table 1. Alternate definitions for ANC content proportion

Name	N data points (country-years)	Logit coefficient	Logit coefficient SD
anc_bin_abce	3	0.182	0.003
anc_bin_abde	2	0.032	0.004
anc_bin_acde	640	0.082	0.005
anc_bin_bcde	217	0.457	0.006
anc_bin_abcde*	1550	0.000	0.000

Notes: Coefficient captures the difference between the alternative definition versus the reference definition. *Reference definition

Supplementary Figure 10a. Matched pairs of complete gold standard measures and unadjusted partial alternative measures of ANC content proportion

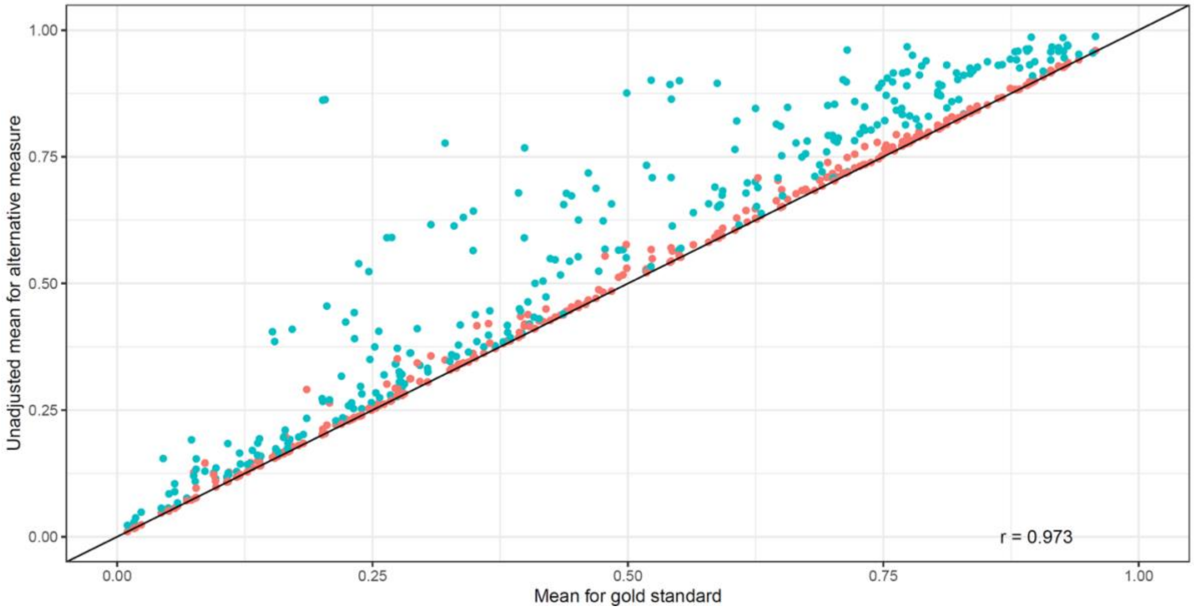

Supplementary Figure 10b. Matched pairs of complete gold standard measures and adjusted partial alternative measures of ANC content proportion

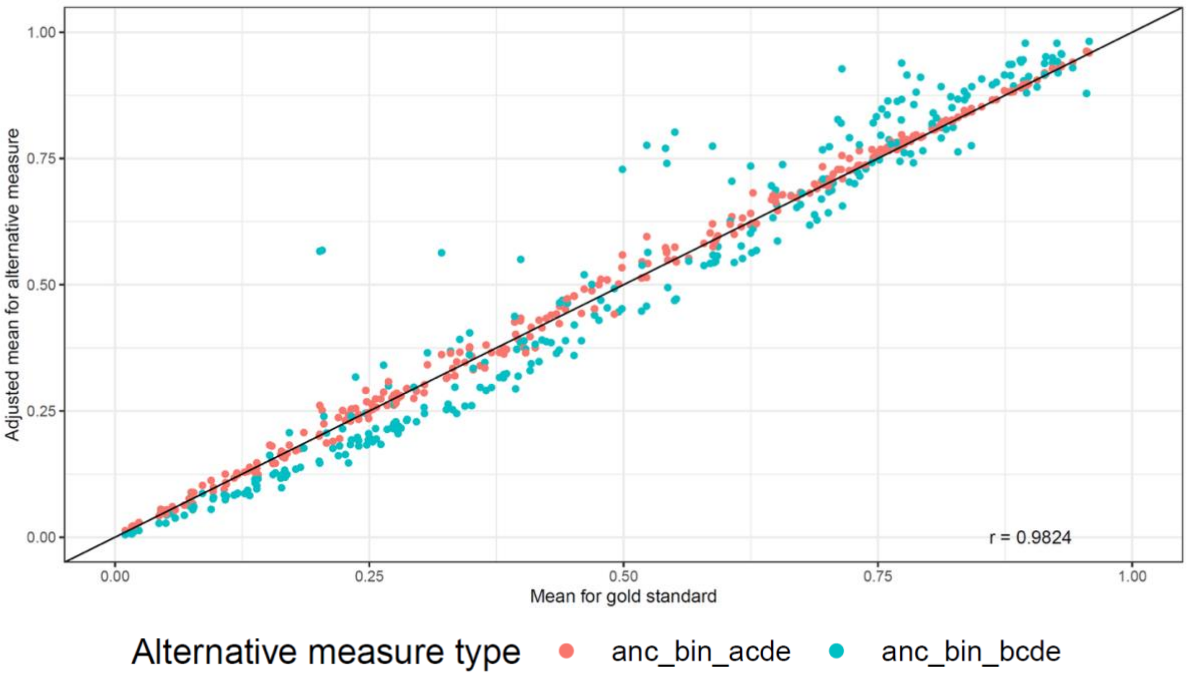

Supplementary Figure 11. Original and adjusted mean ANC content proportion by definition

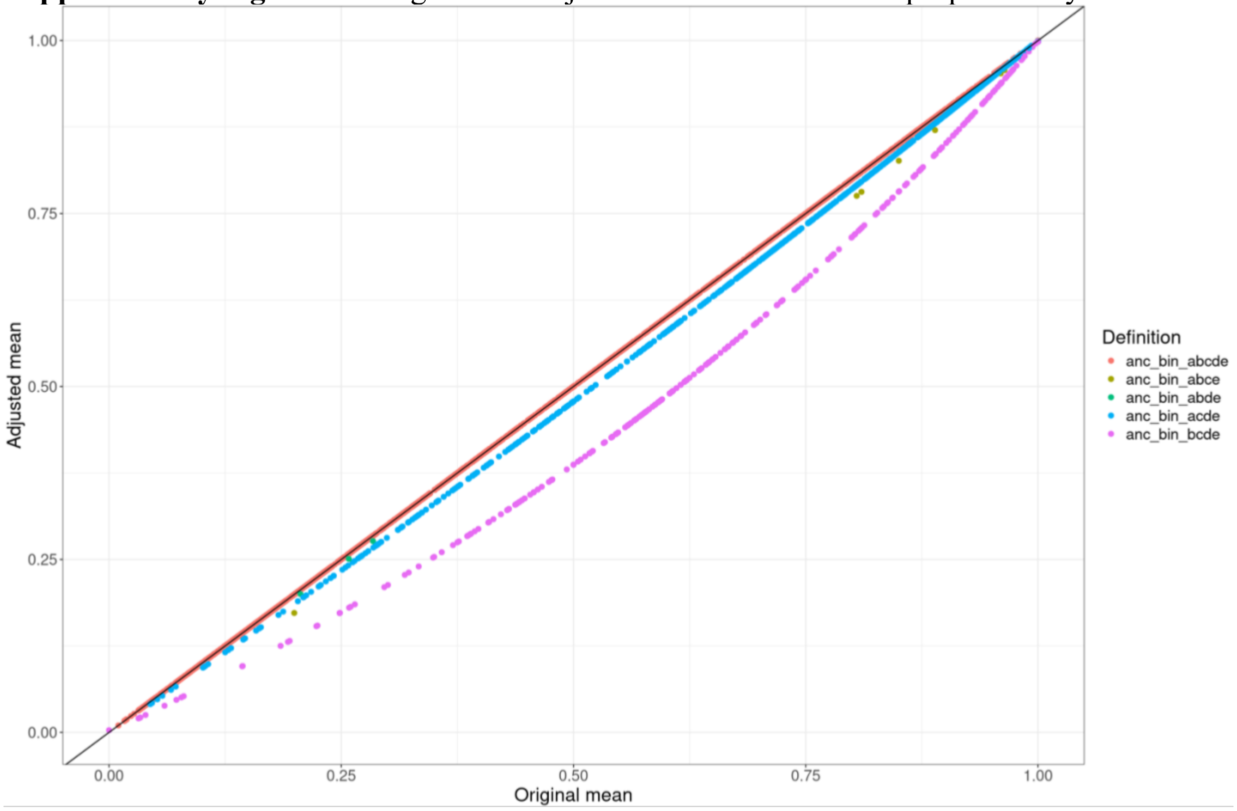

Supplementary Table 2. Alternate definitions for ANC content mean

Name	N data points	Logit coefficient	Logit coefficient SD
anc_mean_abce	3	0.149	0.002
anc_mean_abde	2	-0.157	0.003
anc_mean_acde	640	-0.166	0.003
anc_mean_bcde	217	-0.016	0.004
anc_mean_abcde*	1550	0.000	0.000

Notes: Coefficient captures the difference between the alternative definition mean number versus the reference definition mean number received. *Reference definition

Supplementary Figure 12a. Matched pairs of complete gold standard measures and unadjusted partial alternative measures of ANC content mean

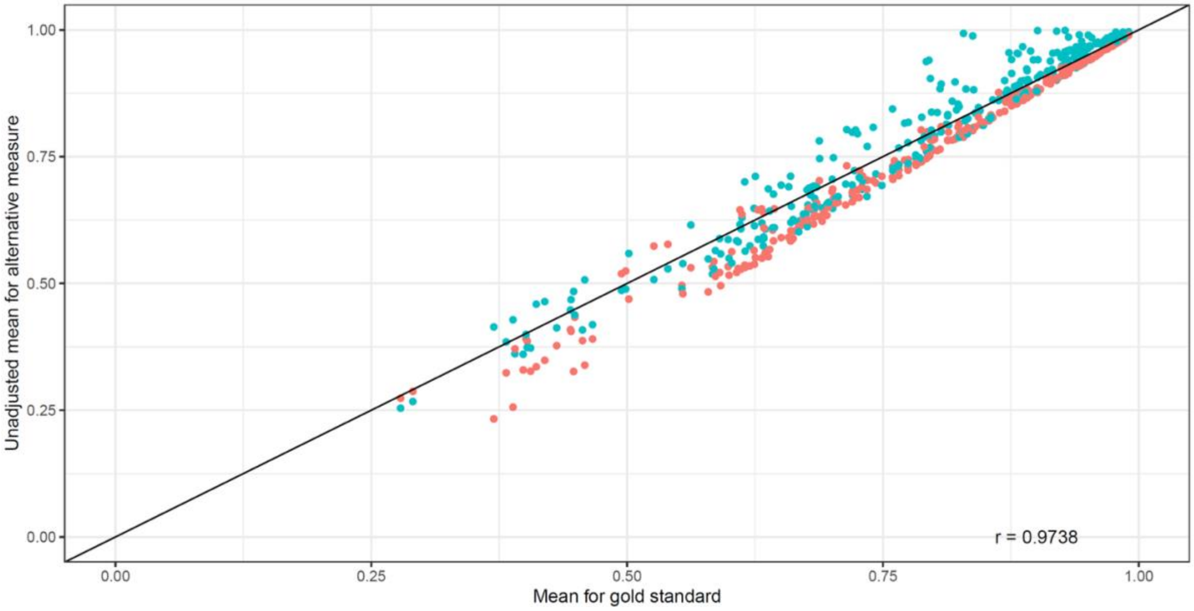

Supplementary Figure 12b. Matched pairs of complete gold standard measures and adjusted partial alternative measures of ANC content mean

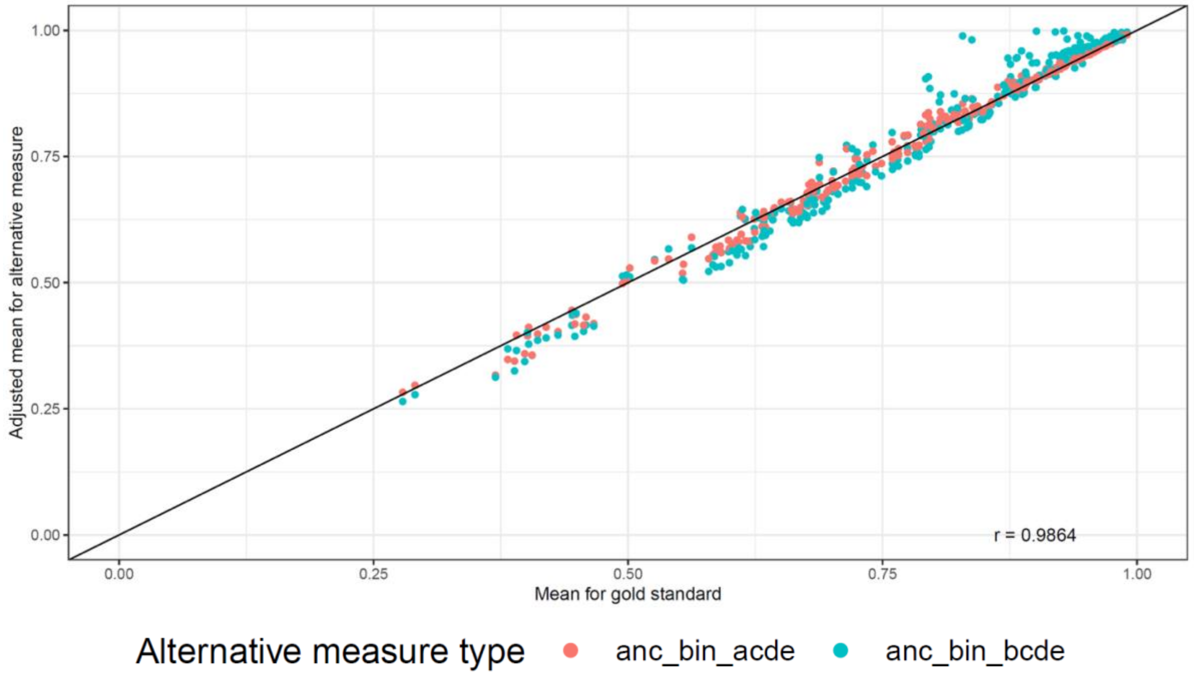

Supplementary Figure 13. Original and adjusted mean ANC content mean by definition

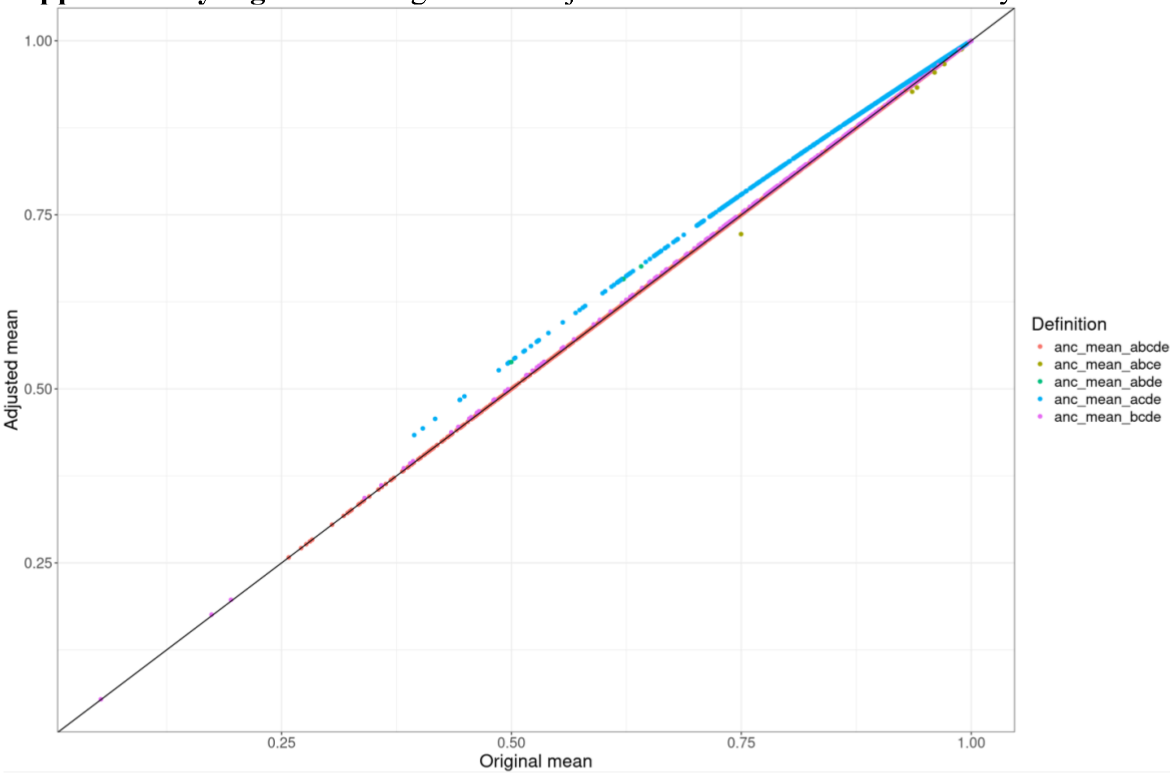

Modelling approach

Supplementary Figure 14. Modeling strategy flowchart

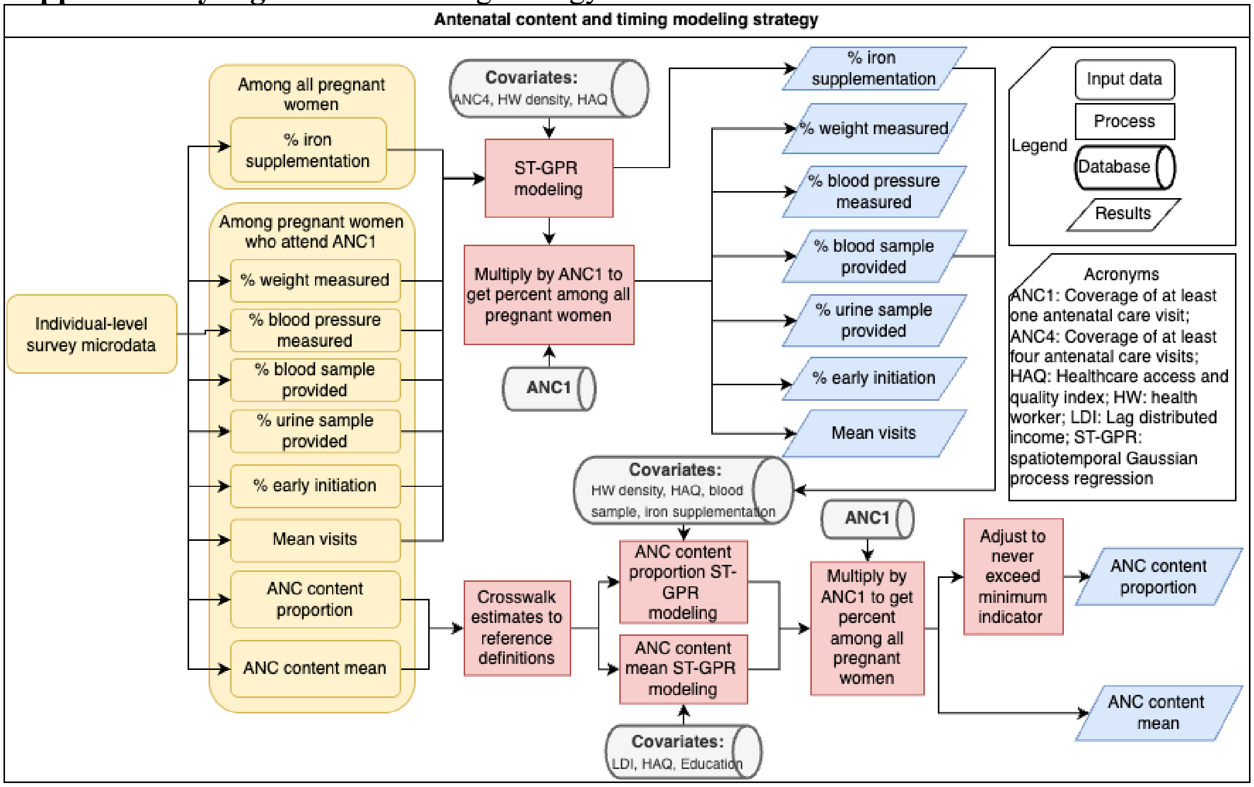

Each antenatal care content and timing indicator was modeled separately using spatiotemporal Gaussian process regression (ST-GPR) to produce a complete time series of estimates for every location in the analysis for the period of 1995 to 2023.[3] ST-GPR is a three-stage process that leverages relationships between data and predictive covariates and borrows strength across geography and time. It is a tool used frequently in the Global Burden of Disease, Risk Factors and Injuries (GBD) study and was chosen because it allows for a flexible time trend, making use of the large amount of data we have on ANC content and timing. The predicted ANC indicator p in a location l at time t is given by:

$$p_{l,t} = g_l(t) + \epsilon_{l,t}$$

where $g_l(t) \sim GP(m_l(t),\ Cov(g_l(t)))$ and $\epsilon_{l,t} \sim Normal(0, \sigma_p^2)$.

The derivation of the mean function $m_l(t)$, covariance function, $Cov(g_l(t))$ and error variance, σ_p^2 are described at length in Supplementary appendix 1 of the GBD 2021 Risk Factor study (pp. 32-36)[3] and summarized below.

In the first step, we estimated a linear mixed effects regression using the covariates defined Supplementary Table 4, which were selected through lasso regression, and nested random effects at the super-region, region and country levels, using the GBD location hierarchy.[4] Coefficients

from the linear models are included in Supplementary tables 5 and 6. From these models, we created a first set of predicted estimates for all locations and years.

Second, we used space and time weights to smooth the residuals between the data and the predictions from the linear mixed effects model over geography and time, producing an updated time series for each location. This step allows us to borrow information from locations and time periods with a lot of data and inform areas of data sparsity. The mean function $m_l(t)$ is expressed as $logit(p_l(t) = X_c\beta + h(r_{c,t})$, where $X\beta$ is the summation of the components of the hierarchical mixed-effects regression from the first stage and $h(r_{l,t})$ is the smoothing function for the residuals.

Finally, Gaussian process regression improves predictions for countries and years that have input data available and incorporates uncertainty in the form of draws. The covariance function $Cov(g_l(t))$ is a Matern-Euclidian covariance function that incorporates information on the deviation between the first-stage regression estimates and the second-stage spatiotemporal smoothing step. We sampled 1000 draws from the Gaussian process regression, using the mean as the reported estimates and the 2.5 and 97.5 draws to estimate the uncertainty interval.

Because the ANC content proportion indicator is defined as the proportion of women who received all five indicators, its value should never exceed minimum value of the component indicators. After modeling, we enforced this relationship by adjusting the content proportion to be the minimum value among the five indicators if it exceeded any indicator. Supplementary Figure 15 shows the extent of this adjustment in 2023.

Supplementary Table 4. Definitions of Covariates

Indicator	Definition
Healthcare Access and Quality (HAQ) Index	An index of scaled mortality-to-incidence ratios and risk-standardised death rates for 32 causes of death that should not occur in the presence of timely, quality health care[5]
ANC4	Proportion of pregnant women receiving 4 or more antenatal care visits including 1 or more from a skilled provider[6]
Health worker density	Number of employed health workers (of any specialty) per 10,000 population[7]
Lag distributed income (LDI) per capita	Lag distributed income per capita (international dollars): gross domestic product per capita that has been smoothed over the preceding 10 years[6]
Education per capita	Education (years per capita) aggregated by age (15+) and sex[6]

Supplementary Table 5. ST-GPR stage 1 coefficients for individual outcomes

	Weight	Iron supplementation	Urine sample	Blood sample	Blood pressure	Early initiation	Mean visits
Intercept	-0.498 (0.587)	-2.015 (0.462)	-3.474 (0.617)	-3.49 (0.678)	-1.183 (0.761)	-1.509 (0.193)	0.384 (0.298)
ANC4	1.583 (0.398)	3.422 (0.159)	2.351 (0.309)	3.108 (0.378)	2.812 (0.431)	0.751 (0.109)	5.602 (0.135)

HW density	0.001 (0.001)	0.000 (0.000)	0.003 (0.001)	0.006 (0.001)	0.005 (0.001)	0.001 (0.000)	0.006 (0.000)
HAQ index	0.083 (0.012)	0.024 (0.005)	0.107 (0.009)	0.088 (0.011)	0.073 (0.012)	0.033 (0.004)	0.017 (0.004)

Coefficient (Standard Error)

Supplementary Table 6. ST-GPR stage 1 coefficients for composite outcomes

	ANC content proportion	ANC content mean
Intercept	-5.058 (0.184)	0.250 (0.091)
Iron supplementation	1.633 (0.110)	
Blood sample	4.069 (0.099)	
Health worker density	0.000 (0.000)	
HAQ index	0.030 (0.003)	0.006 (0.004)
LDI per capita		0.000 (0.000)
Education per capita		0.133 (0.017)

Coefficient (Standard Error)

Supplementary Figure 15. Post-modeling adjustment of ANC content proportion

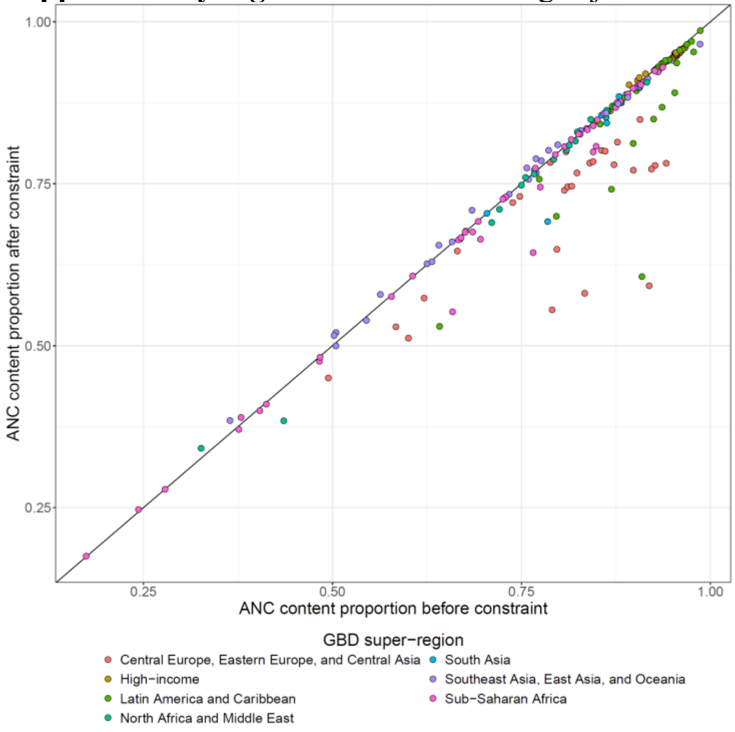

Any Antenatal Care Attendance Estimation

Attendance of any antenatal care (ANC1) was estimated separately as part of the GBD 2023 covariate database. As it is used throughout the estimation process of our indicators and presented in Table 2 as a point of comparison for the content and timing indicators, we summarize the methods used to estimate this indicator here.

ANC1 is defined as the proportion of women with a live birth that receive at least one antenatal care visit from a skilled provider (doctor, nurse, midwife, or country equivalent) during pregnancy. Data on this indicator was identified using the same repository of data sources as the content and timing indicators, and estimates were also modelled using ST-GPR. In the first stage of ST-GPR, a set of predictive covariates from the GBD covariates database were identified and mixed-effects linear regressions were run for every combination of these covariates and the input data. An ensemble prediction was then created which combined these individual models by weighing each sub-model inversely proportional to its out-of-sample root-mean-squared error. Models with any coefficients that were not statistically significant or with effect sizes in the opposite direction from what was expected were excluded. The second and third stages of ST-GPR were the same as those for the content and timing indicators.

Analysis

We sought to understand whether the ANC content and timing indicators were more predictive of health outcomes than coverage of at least four ANC visits (ANC4), which historically has been the most widely estimated and reported measure of ANC. ANC4 is currently used as a covariate in the GBD for modeling maternal and neonatal disorders.[8] We used GBD 2023 estimates of the maternal mortality ratio (MMR), neonatal mortality rate (NMR) and stillbirth rate (SBR) for all country-years in our study to compare the predictive power of ANC4 versus the measures of ANC content we developed. Our focus was on the ANC content mean indicator as it captures the most variation in ANC content as compared to the ANC content proportion, which is constrained by the indicator received by the lowest share of women; the individual ANC content and timing indicators also represent a more limited set of features of ANC.

We focused on within-country variation, removing time-invariant factors from our analysis. We did this by de-meaning all variables in the analysis by subtracting the values of all variables from the mean value across all years within a country. This is mathematically equivalent to including a fixed effect on country. We took this approach to use Rover, a covariate selection tool developed for the GBD, that tests all combinations of covariates and ranks them as predictors in a Bayesian modeling average framework using out-of-sample root mean squared error (OOS RMSE).[9] Rover cannot accommodate fixed effects on country.

We identified a set of non-ANC related covariates drawn from the GBD covariate database that minimized the out of sample root mean squared error (OOS RMSE), with all candidates listed in Supplementary Table 7. Using linear regression in Rover, we used all possible combinations of the covariates to predict the three outcomes, then selected the models in which the coefficients were in the expected direction listed in Supplementary Table 7. Of the models that met these criteria, we identified the model that minimized OOS RMSE holding out 20% of countries. The

predictors selected in this model were then used throughout the rest of the analyses described below. The selected predictors for NMR were: the healthcare access and quality index (HAQI), facility delivery rate, % of births among women older than age 35, and the age-specific fertility rate for adolescents aged 10-19. The selected predictors used for MMR were: HAQI, skilled birth attendance rate, % of births over age 35, the HIV mortality rate for females aged 10-54, and hospital beds per capita. The selected predictors used for SBR were: maternal education, skilled birth attendance rate, socio-demographic index, hospital beds per capita and the age-specific fertility rate for adolescents aged 10-19.

Next, we compared the variance in the outcomes that were explained by ANC4 versus the ANC mean content indicator. These regressions are shown in Supplementary Table 11. The r-squared was higher for models with the ANC content mean as compared to those that included ANC4. Together with the other selected predictors, the r-squared of the NMR model with ANC4 was 77.9%, while the NMR model controlling instead for ANC mean content had an r-squared of 79.5%. Similarly, the model with ANC4 explained 39.7% of the variance of MMR while the MMR model with ANC mean content explained 42.2%. The respective values for SBR were 57.1% versus 57.7%. To better understand the respective contributions to explained variation from ANC4 versus the ANC content mean, we decomposed the relative contribution of each to the outcomes using Shapley value decomposition.[10] Using this method, ANC4 contributed to 17.9% (95% Uncertainty interval (UI): 17.0-18.7) of the variance in NMR while ANC mean content contributed 22.7% (21.2-23.6). ANC4 explained 8.3% (7.3-9.3) of the information on MMR while ANC mean content contributed 15.3% (13.6-16.9). ANC4 explained 9% (9-10) of the information on SBR while ANC mean content contributed 12% (11-13), see Supplementary Table 12. Thus, the ANC content mean both increased model performance overall and itself contributes more to variation in the health outcomes relative to ANC4.

As a final step, we assessed whether additional ANC content and timing predictors would further reduce OOS RMSE along with the previously selected covariates. Using linear regressions, we modeled the outcomes using all combinations of the ANC indicators described in the main paper: iron supplementation, weight measured, blood pressure measured, blood sample, urine sample, ANC content mean, early ANC initiation and mean ANC visits. We selected the models where coefficients were inversely associated with the outcome, consistent with theoretical expectations for their impact on NMR and MMR. We then identified the model with the lowest OOS RMSE. These regressions are shown in Supplementary Table 11. The ANC indicators that minimized OOS RMSE for NMR in addition to the ANC content mean were: blood pressure measured and weight measured. The ANC content mean indicator alone minimized OOS RMSE for the MMR – no additional covariates reduced OOS RMSE. Blood pressure measurement minimized OOS RMSE for SBR.

Because there is a lag between ANC provision and the health outcomes, we conducted a sensitivity analysis where we lagged the ANC indicators by one year in the regressions. The results were robust to this specification.

Supplementary Table 7. Covariates considered in health outcomes analysis

Covariate	Definition	Direction
Lag-distributed income (LDI) per capita	Gross domestic product per capita that has been smoothed over the preceding 10 years[6]	-

TFR	Total Fertility Rate[11]	+
Maternal Education	Mean number of years of education among women 15-49[12]	-
Education per capita	Education (years per capita) aggregated by age (15+) and sex[12]	-
Healthcare Access and Quality (HAQ) Index	An index of scaled mortality-to-incidence ratios and risk-standardised death rates for 32 causes of death that should not occur in the presence of timely, quality health care[5]	-
Socio-demographic index (SDI)	A measure of development estimated via principal component analysis using log-transformed LDI, TFR (<25), and education years per capita over age 15[9]	-
Facility delivery rate	Percent of women giving birth in a health facility[6]	-
Skilled birth attendant (SBA)	Percent of women giving birth with a skilled birth attendant (mainly nurses, doctors, midwives)[6]	-
HIV mortality	HIV mortality for females aged 10-54[8]	+
% births over 35	Proportion of live births by mothers age 35 and older[11]	None
% births over 40	Proportion of live births by mothers age 40 and older[11]	None
Adolescent ASFR	Age-specific fertility rate in adolescents, ages 10 - 19[11]	+
Hospital beds	Hospital beds per 1000 people[6]	-

Location codes

Supplementary Table 8. Location codes for countries in Figure 4

Code	Country	Region
ALB	Albania	Central Europe, Eastern Europe, and Central Asia
ARM	Armenia	Central Europe, Eastern Europe, and Central Asia
AZE	Azerbaijan	Central Europe, Eastern Europe, and Central Asia
BIH	Bosnia and Herzegovina	Central Europe, Eastern Europe, and Central Asia
BLR	Belarus	Central Europe, Eastern Europe, and Central Asia
GEO	Georgia	Central Europe, Eastern Europe, and Central Asia
KAZ	Kazakhstan	Central Europe, Eastern Europe, and Central Asia
KGZ	Kyrgyzstan	Central Europe, Eastern Europe, and Central Asia
MDA	Republic of Moldova	Central Europe, Eastern Europe, and Central Asia
MKD	North Macedonia	Central Europe, Eastern Europe, and Central Asia
MNE	Montenegro	Central Europe, Eastern Europe, and Central Asia
MNG	Mongolia	Central Europe, Eastern Europe, and Central Asia
SRB	Serbia	Central Europe, Eastern Europe, and Central Asia
TJK	Tajikistan	Central Europe, Eastern Europe, and Central Asia
TKM	Turkmenistan	Central Europe, Eastern Europe, and Central Asia
UKR	Ukraine	Central Europe, Eastern Europe, and Central Asia
UZB	Uzbekistan	Central Europe, Eastern Europe, and Central Asia
ARG	Argentina	Latin America and Caribbean
BLZ	Belize	Latin America and Caribbean
BOL	Bolivia (Plurinational State of)	Latin America and Caribbean
BRA	Brazil	Latin America and Caribbean
COL	Colombia	Latin America and Caribbean
CRI	Costa Rica	Latin America and Caribbean
CUB	Cuba	Latin America and Caribbean
DMA	Dominica	Latin America and Caribbean
DOM	Dominican Republic	Latin America and Caribbean
ECU	Ecuador	Latin America and Caribbean
GRD	Grenada	Latin America and Caribbean
GTM	Guatemala	Latin America and Caribbean
HND	Honduras	Latin America and Caribbean
HTI	Haiti	Latin America and Caribbean
JAM	Jamaica	Latin America and Caribbean

LCA	Saint Lucia	Latin America and Caribbean
MEX	Mexico	Latin America and Caribbean
NIC	Nicaragua	Latin America and Caribbean
PER	Peru	Latin America and Caribbean
PRY	Paraguay	Latin America and Caribbean
SLV	El Salvador	Latin America and Caribbean
SUR	Suriname	Latin America and Caribbean
VCT	Saint Vincent and the Grenadines	Latin America and Caribbean
VEN	Venezuela (Bolivarian Republic of)	Latin America and Caribbean
AFG	Afghanistan	North Africa and Middle East
DZA	Algeria	North Africa and Middle East
EGY	Egypt	North Africa and Middle East
IRN	Iran (Islamic Republic of)	North Africa and Middle East
IRQ	Iraq	North Africa and Middle East
JOR	Jordan	North Africa and Middle East
LBN	Lebanon	North Africa and Middle East
LBY	Libya	North Africa and Middle East
MAR	Morocco	North Africa and Middle East
PSE	Palestine	North Africa and Middle East
SDN	Sudan	North Africa and Middle East
SYR	Syrian Arab Republic	North Africa and Middle East
TUN	Tunisia	North Africa and Middle East
TUR	Türkiye	North Africa and Middle East
YEM	Yemen	North Africa and Middle East
BGD	Bangladesh	South Asia
BTN	Bhutan	South Asia
IND	India	South Asia
NPL	Nepal	South Asia
PAK	Pakistan	South Asia
CHN	China	Southeast Asia, East Asia, and Oceania
FJI	Fiji	Southeast Asia, East Asia, and Oceania
FSM	Micronesia (Federated States of)	Southeast Asia, East Asia, and Oceania
IDN	Indonesia	Southeast Asia, East Asia, and Oceania
KHM	Cambodia	Southeast Asia, East Asia, and Oceania
KIR	Kiribati	Southeast Asia, East Asia, and Oceania

LAO	Lao People's Democratic Republic	Southeast Asia, East Asia, and Oceania
LKA	Sri Lanka	Southeast Asia, East Asia, and Oceania
MDV	Maldives	Southeast Asia, East Asia, and Oceania
MHL	Marshall Islands	Southeast Asia, East Asia, and Oceania
MMR	Myanmar	Southeast Asia, East Asia, and Oceania
MUS	Mauritius	Southeast Asia, East Asia, and Oceania
MYS	Malaysia	Southeast Asia, East Asia, and Oceania
PHL	Philippines	Southeast Asia, East Asia, and Oceania
PNG	Papua New Guinea	Southeast Asia, East Asia, and Oceania
PRK	Democratic People's Republic of Korea	Southeast Asia, East Asia, and Oceania
SLB	Solomon Islands	Southeast Asia, East Asia, and Oceania
THA	Thailand	Southeast Asia, East Asia, and Oceania
TLS	Timor-Leste	Southeast Asia, East Asia, and Oceania
TON	Tonga	Southeast Asia, East Asia, and Oceania
TUV	Tuvalu	Southeast Asia, East Asia, and Oceania
VNM	Viet Nam	Southeast Asia, East Asia, and Oceania
VUT	Vanuatu	Southeast Asia, East Asia, and Oceania
WSM	Samoa	Southeast Asia, East Asia, and Oceania
AGO	Angola	Sub-Saharan Africa
BDI	Burundi	Sub-Saharan Africa
BEN	Benin	Sub-Saharan Africa
BFA	Burkina Faso	Sub-Saharan Africa
BWA	Botswana	Sub-Saharan Africa
CAF	Central African Republic	Sub-Saharan Africa
CIV	Côte d'Ivoire	Sub-Saharan Africa
CMR	Cameroon	Sub-Saharan Africa
COD	Democratic Republic of the Congo	Sub-Saharan Africa
COG	Congo	Sub-Saharan Africa
COM	Comoros	Sub-Saharan Africa
CPV	Cabo Verde	Sub-Saharan Africa
DJI	Djibouti	Sub-Saharan Africa
ERI	Eritrea	Sub-Saharan Africa
ETH	Ethiopia	Sub-Saharan Africa
GAB	Gabon	Sub-Saharan Africa
GHA	Ghana	Sub-Saharan Africa

GIN	Guinea	Sub-Saharan Africa
GMB	Gambia	Sub-Saharan Africa
GNB	Guinea-Bissau	Sub-Saharan Africa
GNQ	Equatorial Guinea	Sub-Saharan Africa
KEN	Kenya	Sub-Saharan Africa
LBR	Liberia	Sub-Saharan Africa
LSO	Lesotho	Sub-Saharan Africa
MDG	Madagascar	Sub-Saharan Africa
MLI	Mali	Sub-Saharan Africa
MOZ	Mozambique	Sub-Saharan Africa
MRT	Mauritania	Sub-Saharan Africa
MWI	Malawi	Sub-Saharan Africa
NAM	Namibia	Sub-Saharan Africa
NER	Niger	Sub-Saharan Africa
NGA	Nigeria	Sub-Saharan Africa
RWA	Rwanda	Sub-Saharan Africa
SEN	Senegal	Sub-Saharan Africa
SLE	Sierra Leone	Sub-Saharan Africa
SOM	Somalia	Sub-Saharan Africa
SSD	South Sudan	Sub-Saharan Africa
STP	Sao Tome and Principe	Sub-Saharan Africa
SWZ	Eswatini	Sub-Saharan Africa
TCD	Chad	Sub-Saharan Africa
TGO	Togo	Sub-Saharan Africa
TZA	United Republic of Tanzania	Sub-Saharan Africa
UGA	Uganda	Sub-Saharan Africa
ZAF	South Africa	Sub-Saharan Africa
ZMB	Zambia	Sub-Saharan Africa
ZWE	Zimbabwe	Sub-Saharan Africa

Part 2. Supplementary results

Maps of individual ANC content indicators, 2023

Supplementary Figure 16. Proportion who had their weight measured during their ANC visit among women with a live birth, 2023

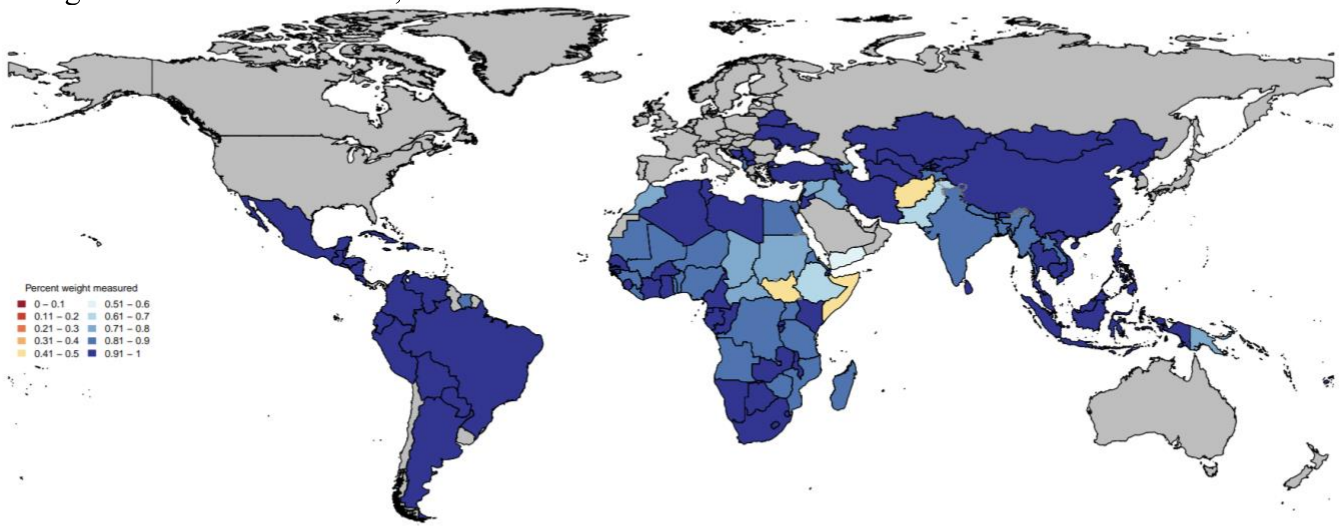

Supplementary Figure 17. Proportion who received iron supplementation during their ANC visit among women with a live birth, 2023

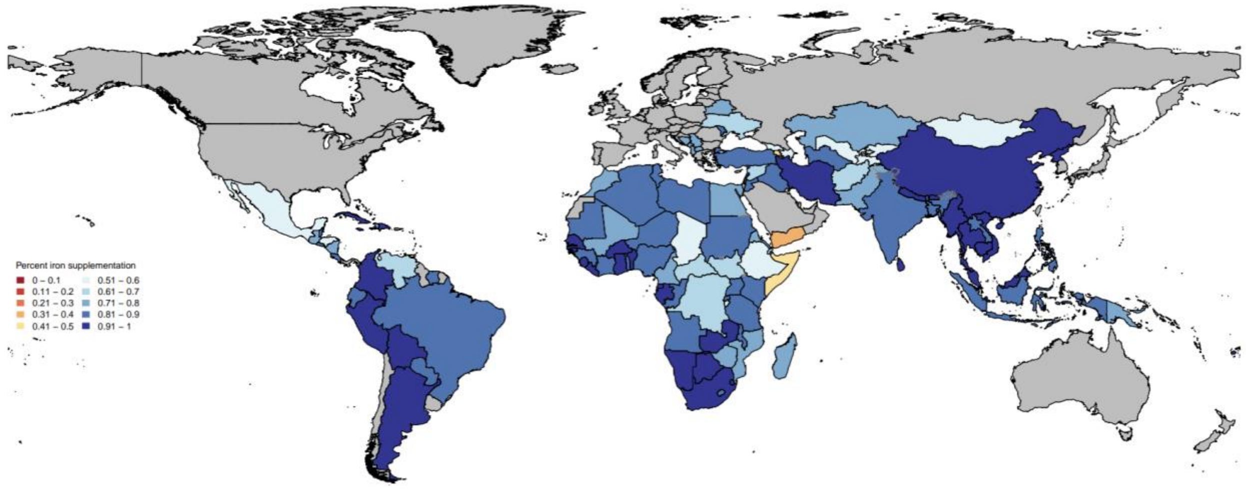

Supplementary Figure 18. Proportion who provided a urine sample during their ANC visit among women with a live birth, 2023

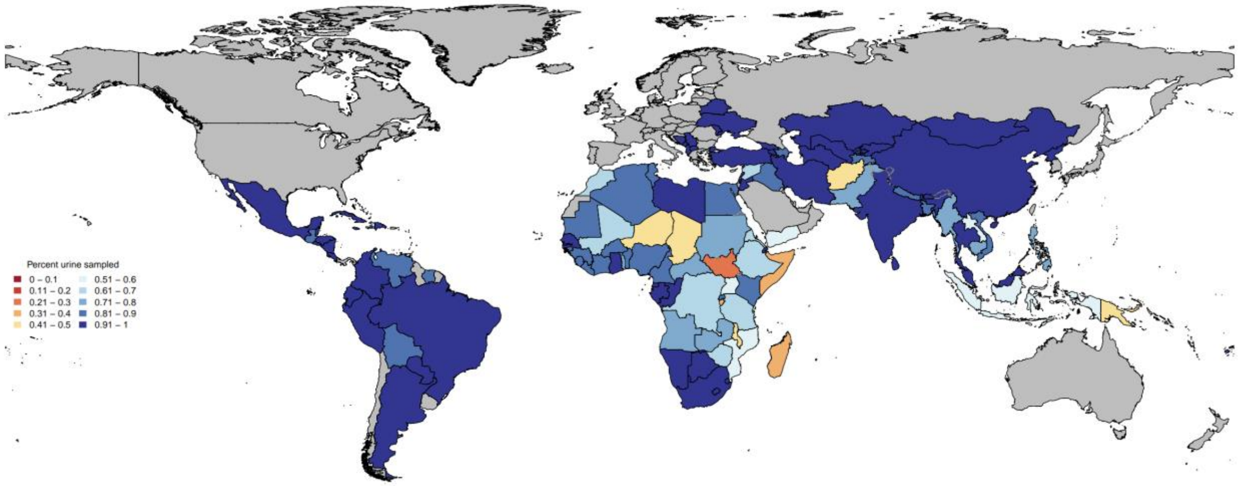

Supplementary Figure 19. Proportion who had a blood sample taken during their ANC visit among women with a live birth, 2023

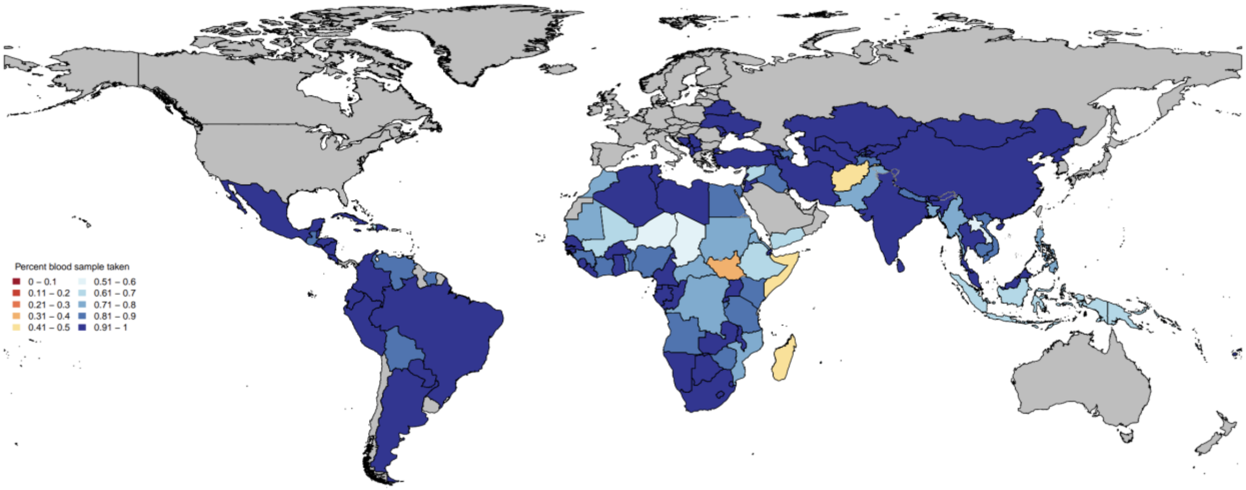

Supplementary Figure 20. Proportion who had blood pressure measured during their ANC visit among women with a live birth, 2023

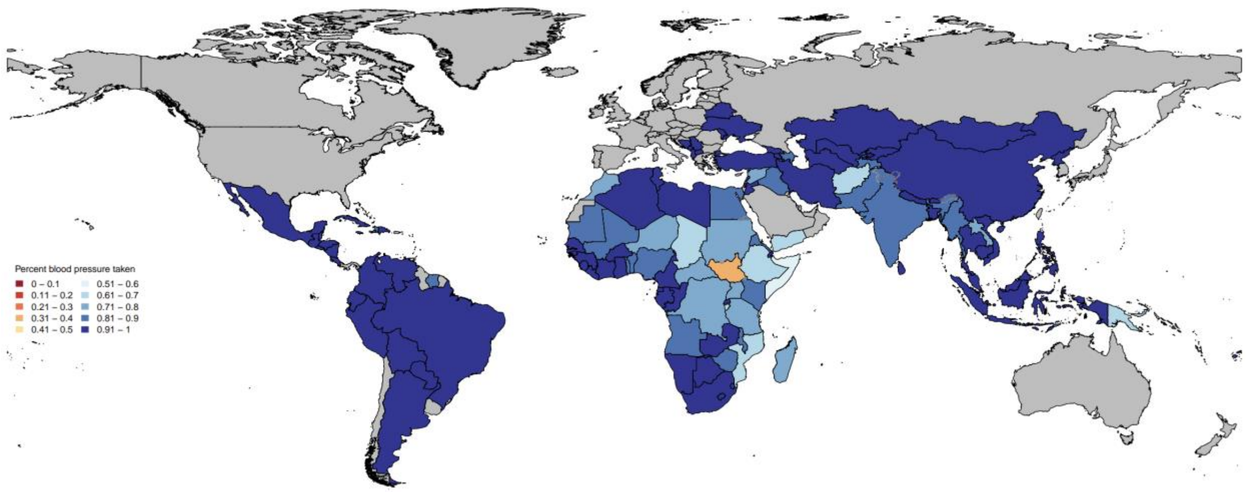

Tables presenting change over time in ANC content and timing indicators, 1995-2023

Supplementary Table 9. Absolute difference in coverage indicators by country, 1995-2023

	Urine sample	Weight measured	Blood sample	Blood pressure measured	Iron supplementation	ANC content proportion	Early ANC initiation	Mean ANC visits
Afghanistan	0.43	0.41	0.38	0.48	0.36	0.26	0.17	2.33
Albania	0.46	0.50	0.56	0.48	0.56	0.61	0.20	4.15
Algeria	0.73	0.41	0.72	0.56	0.43	0.73	0.19	3.15
Angola	0.55	0.31	0.25	0.49	0.35	0.46	0.21	3.30
Argentina	0.03	0.01	0.02	0.02	0.05	0.04	0.10	1.77
Armenia	0.09	0.14	0.08	0.08	0.27	0.28	0.37	3.43
Azerbaijan	0.44	0.46	0.42	0.36	0.32	0.36	0.17	2.85
Bangladesh	0.69	0.69	0.72	0.70	0.48	0.64	0.23	2.63
Belarus	0.00	0.00	0.00	0.00	0.10	0.10	0.13	2.29
Belize	0.09	0.06	0.06	0.06	0.09	0.12	0.15	2.46
Benin	0.27	0.13	0.63	0.15	0.13	0.62	0.30	0.61
Bhutan	0.60	0.49	0.54	0.42	0.27	0.57	0.31	4.14
Bolivia (Plurinational State of)	0.66	0.40	0.71	0.41	0.48	0.72	0.19	3.03
Bosnia and Herzegovina	0.12	0.13	0.12	0.11	0.22	0.23	0.15	2.34
Botswana	0.15	0.07	0.09	0.07	0.11	0.17	0.24	2.07
Brazil	0.20	0.13	0.23	0.12	0.20	0.28	0.14	3.08
Burkina Faso	0.47	0.39	0.76	0.37	0.21	0.68	0.20	2.17
Burundi	0.35	0.13	0.79	0.35	0.06	0.16	0.39	2.58
Cabo Verde	0.10	0.05	0.15	0.05	0.10	0.14	0.21	2.82
Cambodia	0.73	0.71	0.83	0.73	0.72	0.70	0.61	4.94

	Urine sample	Weight measured	Blood sample	Blood pressure measured	Iron supplementation	ANC content proportion	Early ANC initiation	Mean ANC visits
Cameroon	0.32	0.19	0.34	0.22	0.15	0.32	0.08	1.04
Central African Republic	0.22	0.13	0.30	0.19	0.25	0.35	0.09	0.62
Chad	0.33	0.41	0.40	0.42	0.34	0.33	0.09	1.48
China	0.47	0.24	0.44	0.22	0.34	0.50	0.30	5.44
Colombia	0.28	0.15	0.26	0.14	0.33	0.41	0.14	2.66
Comoros	0.37	0.16	0.34	0.24	0.18	0.44	0.25	1.75
Congo	0.16	0.09	0.10	0.12	0.31	0.33	0.14	1.73
Costa Rica	0.09	0.03	0.03	0.04	0.03	0.05	0.07	1.20
Cuba	0.01	0.01	0.01	0.01	0.00	0.01	0.06	2.94
Côte d'Ivoire	0.33	0.18	0.52	0.23	0.24	0.54	0.16	1.52
Democratic People's Republic of Korea	0.09	0.01	0.07	0.01	0.04	0.07	0.12	0.70
Democratic Republic of the Congo	0.44	0.19	0.45	0.34	0.33	0.50	0.09	1.69
Djibouti	0.25	0.13	0.26	0.13	0.30	0.32	0.15	1.77
Dominica	0.02	0.02	0.02	0.01	0.02	0.03	0.09	2.54
Dominican Republic	0.11	0.06	0.11	0.04	0.06	0.15	0.11	2.97
Ecuador	0.48	0.18	0.53	0.20	0.27	0.61	0.17	2.71
Egypt	0.68	0.59	0.69	0.62	0.63	0.68	0.14	6.35
El Salvador	0.32	0.23	0.31	0.21	0.20	0.32	0.15	1.92
Equatorial Guinea	0.71	0.41	0.52	0.58	0.56	0.66	0.44	6.58
Eritrea	0.52	0.36	0.55	0.42	0.39	0.54	0.16	2.75
Eswatini	0.13	0.05	0.10	0.08	0.05	0.11	0.08	1.19
Ethiopia	0.57	0.50	0.58	0.52	0.42	0.40	0.22	3.02
Fiji	0.09	0.01	0.08	0.02	0.05	0.09	0.05	1.30
Gabon	0.11	0.05	0.14	0.08	0.12	0.16	0.20	1.90
Gambia	0.23	0.10	0.20	0.11	0.07	0.21	0.17	1.42
Georgia	0.11	0.11	0.11	0.10	0.21	0.25	0.12	1.85
Ghana	0.23	0.18	0.24	0.16	0.17	0.33	0.23	2.05
Grenada	0.03	0.02	0.03	0.01	0.05	0.05	0.11	2.75
Guatemala	0.64	0.28	0.70	0.54	0.51	0.70	0.11	2.26
Guinea	0.56	0.34	0.64	0.39	0.23	0.62	0.06	1.70
Guinea-Bissau	0.38	0.13	0.40	0.19	0.16	0.47	0.14	0.97
Haiti	0.40	0.24	0.37	0.24	0.31	0.45	0.04	0.17
Honduras	0.47	0.23	0.47	0.25	0.01	0.27	0.15	2.27
India	0.53	0.49	0.51	0.47	0.34	0.57	0.30	2.27
Indonesia	0.47	0.33	0.61	0.33	0.30	0.48	0.23	3.46
Iran (Islamic Republic of)	0.25	0.21	0.23	0.17	0.14	0.27	0.14	3.13
Iraq	0.58	0.48	0.55	0.40	0.40	0.62	0.26	0.58
Jamaica	0.03	0.02	0.04	0.01	0.03	0.04	0.08	2.15
Jordan	0.22	0.12	0.21	0.11	0.16	0.35	0.10	2.14
Kazakhstan	0.08	0.13	0.08	0.09	0.25	0.27	0.22	0.98
Kenya	0.15	0.04	0.09	0.07	0.25	0.40	0.08	0.78

	Urine sample	Weight measured	Blood sample	Blood pressure measured	Iron supplementation	ANC content proportion	Early ANC initiation	Mean ANC visits
Kiribati	0.26	0.10	0.28	0.16	0.23	0.25	0.04	1.82
Kyrgyzstan	0.01	0.02	0.01	0.01	0.16	0.16	0.12	-0.96
Lao People's Democratic Republic	0.53	0.54	0.56	0.59	0.67	0.51	0.27	4.41
Lebanon	0.26	0.12	0.27	0.19	0.14	0.33	0.15	2.79
Lesotho	0.18	0.03	0.12	0.05	0.38	0.44	0.11	1.20
Liberia	0.63	0.33	0.67	0.37	0.15	0.70	0.19	1.67
Libya	0.22	0.18	0.19	0.15	0.16	0.23	0.11	3.13
Madagascar	0.16	0.37	0.28	0.21	0.38	0.19	0.14	0.80
Malawi	0.24	0.07	0.46	0.13	0.21	0.21	0.16	0.22
Malaysia	0.13	0.01	0.04	0.02	0.05	0.06	0.17	2.38
Maldives	0.39	0.08	0.33	0.16	0.32	0.45	0.13	3.77
Mali	0.42	0.41	0.51	0.43	0.47	0.52	0.03	1.16
Marshall Islands	0.13	0.03	0.11	0.04	0.02	0.11	0.02	0.62
Mauritania	0.63	0.49	0.62	0.51	0.32	0.55	0.19	2.15
Mauritius	-0.03	-0.09	-0.09	-0.09	0.05	-0.05	0.11	1.08
Mexico	0.37	0.15	0.33	0.12	0.03	0.10	0.14	1.78
Micronesia (Federated States of)	0.15	0.05	0.13	0.06	0.06	0.15	0.02	0.57
Mongolia	0.04	0.18	0.05	0.04	0.24	0.25	0.14	1.30
Montenegro	0.10	0.29	0.11	0.24	0.19	0.22	0.07	2.41
Morocco	0.51	0.40	0.58	0.38	0.46	0.54	0.16	3.50
Mozambique	0.35	0.15	0.48	0.13	0.27	0.31	0.00	0.36
Myanmar	0.61	0.22	0.71	0.30	0.13	0.66	0.22	1.83
Namibia	0.15	0.08	0.15	0.15	0.18	0.23	0.19	1.97
Nepal	0.83	0.81	0.81	0.81	0.83	0.81	0.46	3.48
Nicaragua	0.36	0.19	0.38	0.19	0.11	0.28	0.13	1.09
Niger	0.34	0.57	0.44	0.44	0.70	0.34	-0.06	2.22
Nigeria	0.42	0.28	0.42	0.29	0.26	0.47	0.12	0.67
North Macedonia	0.10	0.08	0.09	0.07	0.23	0.24	0.12	2.79
Pakistan	0.64	0.52	0.70	0.65	0.41	0.61	0.19	2.58
Palestine	0.13	0.09	0.27	0.09	0.04	0.10	0.31	4.17
Papua New Guinea	0.19	0.08	0.16	0.12	0.07	0.18	0.05	0.25
Paraguay	0.23	0.10	0.32	0.16	0.21	0.32	0.13	3.63
Peru	0.68	0.32	0.71	0.35	0.59	0.82	0.20	5.24
Philippines	0.43	0.11	0.49	0.08	0.14	0.51	0.22	0.91
Republic of Moldova	0.02	0.06	0.02	0.02	0.08	0.10	0.15	1.01
Rwanda	0.86	0.17	0.88	0.37	0.63	0.71	0.52	1.58
Saint Lucia	0.01	0.02	0.17	0.01	0.06	0.13	0.12	2.43
Saint Vincent and the Grenadines	0.06	0.05	0.06	0.04	0.03	0.07	0.09	2.32
Samoa	0.16	0.04	0.19	0.08	0.17	0.24	0.05	1.83
Sao Tome and Principe	0.15	0.03	0.18	0.03	0.10	0.17	0.20	2.61
Senegal	0.44	0.21	0.66	0.19	0.16	0.69	0.19	2.29
Serbia	0.06	0.07	0.04	0.06	0.21	0.21	0.13	2.02

	Urine sample	Weight measured	Blood sample	Blood pressure measured	Iron supplementation	ANC content proportion	Early ANC initiation	Mean ANC visits
Sierra Leone	0.65	0.37	0.70	0.41	0.21	0.65	0.17	1.87
Solomon Islands	0.28	0.17	0.29	0.20	0.16	0.28	0.03	1.71
Somalia	0.30	0.22	0.28	0.29	0.23	0.12	0.05	0.08
South Africa	0.01	0.01	0.01	0.01	0.03	0.03	0.26	0.56
South Sudan	0.16	0.16	0.23	0.17	0.32	0.18	0.07	1.18
Sri Lanka	0.02	0.02	0.20	0.02	0.02	0.17	0.15	0.99
Sudan	0.61	0.50	0.63	0.58	0.39	0.63	0.24	3.07
Suriname	0.05	0.00	0.06	-0.01	0.20	0.17	0.14	3.02
Syrian Arab Republic	0.56	0.42	0.57	0.39	0.27	0.52	0.17	2.31
Tajikistan	0.23	0.23	0.21	0.17	0.26	0.26	0.16	1.70
Thailand	0.07	0.06	0.07	0.06	0.11	0.16	0.17	2.75
Timor-Leste	0.55	0.29	0.55	0.28	0.55	0.51	0.31	2.44
Togo	0.22	0.03	0.38	0.06	0.13	0.45	0.14	0.92
Tonga	0.08	0.03	0.28	0.17	0.02	0.33	0.01	0.79
Tunisia	0.51	0.22	0.38	0.18	0.37	0.52	0.17	2.98
Turkmenistan	0.03	0.10	0.02	0.02	0.10	0.10	0.15	1.85
Tuvalu	0.40	0.13	0.43	0.22	0.25	0.48	0.10	2.48
Türkiye	0.53	0.56	0.52	0.36	0.33	0.54	0.27	6.13
Uganda	0.43	0.24	0.74	0.28	0.33	0.35	0.17	0.54
Ukraine	0.08	0.08	0.08	0.08	0.18	0.20	0.10	4.29
United Republic of Tanzania	0.25	-0.05	0.22	-0.01	0.27	0.30	0.28	-0.48
Uzbekistan	0.06	0.14	0.07	0.06	0.17	0.18	0.09	1.88
Vanuatu	0.27	0.15	0.29	0.20	0.19	0.35	0.03	1.76
Venezuela (Bolivarian Republic of)	0.14	0.02	0.14	0.02	0.14	0.17	0.06	0.79
Viet Nam	0.77	0.48	0.80	0.65	0.47	0.78	0.27	6.10
Yemen	0.44	0.40	0.43	0.40	0.24	0.24	0.12	1.52
Zambia	0.56	0.10	0.50	0.16	0.22	0.51	0.31	0.31
Zimbabwe	-0.12	-0.03	0.12	0.02	0.16	0.07	0.10	-0.26

Supplementary Table 10. Percent difference in coverage indicators by country, 1995-2023

	Urine sample	Weight measured	Blood sample	Blood pressure measured	Iron supplementation	ANC content proportion	Early ANC initiation	Mean ANC visits
Afghanistan	6.06	4.52	5.99	2.17	1.18	21.28	0.43	2.26
Albania	0.94	1.30	1.45	1.10	3.38	7.62	0.28	1.57
Algeria	5.91	0.75	3.30	1.42	1.03	11.78	0.28	1.16
Angola	2.21	0.58	0.40	1.51	0.72	1.89	0.62	2.09
Argentina	0.03	0.01	0.02	0.02	0.05	0.05	0.13	0.50
Armenia	0.10	0.17	0.09	0.09	1.41	1.63	0.69	0.64
Azerbaijan	0.98	1.32	0.89	0.66	1.60	3.38	0.25	0.86
Bangladesh	5.58	3.89	8.03	3.27	1.43	24.40	0.64	3.06
Belarus	0.00	0.00	0.00	0.00	0.15	0.14	0.16	0.27
Belize	0.10	0.07	0.06	0.07	0.11	0.16	0.29	0.35

	Urine sample	Weight measured	Blood sample	Blood pressure measured	Iron supplementation	ANC content proportion	Early ANC initiation	Mean ANC visits
Benin	0.45	0.17	2.82	0.20	0.16	3.50	1.03	0.16
Bhutan	1.70	1.10	1.27	0.78	0.39	1.91	0.70	2.03
Bolivia (Plurinational State of)	3.11	0.76	4.18	0.77	1.13	9.29	0.32	0.96
Bosnia and Herzegovina	0.14	0.16	0.13	0.12	0.40	0.41	0.19	0.35
Botswana	0.18	0.07	0.10	0.08	0.14	0.26	0.75	0.31
Brazil	0.26	0.15	0.30	0.14	0.28	0.47	0.20	0.52
Burkina Faso	1.16	0.67	4.67	0.67	0.27	5.08	0.58	1.12
Burundi	10.05	0.16	9.98	1.19	0.13	16.73	2.26	1.75
Cabo Verde	0.11	0.05	0.18	0.06	0.11	0.18	0.41	0.62
Cambodia	13.07	2.72	13.54	2.91	2.79	34.73	2.33	5.10
Cameroon	0.56	0.25	0.61	0.31	0.23	0.75	0.20	0.30
Central African Republic	0.42	0.20	0.70	0.34	0.60	1.77	0.30	0.22
Chad	2.39	1.26	3.04	1.45	1.29	5.27	0.24	1.16
China	0.92	0.33	0.83	0.29	0.54	1.23	0.50	0.87
Colombia	0.40	0.19	0.36	0.17	0.54	0.84	0.20	0.50
Comoros	0.71	0.21	0.60	0.36	0.26	1.46	0.63	0.43
Congo	0.21	0.10	0.11	0.15	0.54	0.66	0.31	0.40
Costa Rica	0.11	0.03	0.03	0.04	0.03	0.06	0.09	0.15
Cuba	0.01	0.01	0.01	0.01	0.00	0.01	0.07	0.21
Côte d'Ivoire	0.61	0.24	1.52	0.32	0.36	3.32	0.58	0.59
Democratic People's Republic of Korea	0.11	0.01	0.07	0.01	0.05	0.08	0.17	0.08
Democratic Republic of the Congo	1.80	0.31	1.51	0.79	0.94	6.07	0.55	0.72
Djibouti	0.37	0.15	0.38	0.16	0.88	1.05	1.05	0.60
Dominica	0.02	0.02	0.02	0.01	0.02	0.04	0.12	0.29
Dominican Republic	0.12	0.06	0.12	0.04	0.06	0.20	0.14	0.42
Ecuador	1.07	0.24	1.33	0.26	0.43	2.70	0.27	0.52
Egypt	3.47	2.01	3.62	2.23	3.59	26.29	0.18	1.94
El Salvador	0.51	0.31	0.47	0.28	0.33	0.69	0.23	0.40
Equatorial Guinea	2.86	0.76	1.17	1.57	1.57	3.44	1.72	5.03
Eritrea	3.08	0.80	3.06	1.05	1.19	10.11	0.69	1.23
Eswatini	0.16	0.06	0.12	0.09	0.06	0.14	0.39	0.27
Ethiopia	9.23	2.66	7.50	3.47	2.94	33.21	1.06	3.57
Fiji	0.11	0.01	0.09	0.02	0.05	0.13	0.12	0.20
Gabon	0.12	0.06	0.18	0.09	0.15	0.21	0.39	0.48
Gambia	0.32	0.11	0.26	0.13	0.07	0.30	0.59	0.37
Georgia	0.12	0.12	0.12	0.12	0.57	0.75	0.16	0.33
Ghana	0.31	0.22	0.32	0.19	0.22	0.58	0.57	0.42
Grenada	0.03	0.02	0.03	0.01	0.05	0.06	0.15	0.37
Guatemala	3.13	0.42	5.43	1.38	1.95	17.17	0.17	0.43
Guinea	1.74	0.60	2.76	0.72	0.34	3.74	0.14	0.52
Guinea-Bissau	0.75	0.16	0.80	0.25	0.21	1.39	0.43	0.25

	Urine sample	Weight measured	Blood sample	Blood pressure measured	Iron supplementation	ANC content proportion	Early ANC initiation	Mean ANC visits
Haiti	1.37	0.42	1.21	0.41	0.65	3.03	0.08	0.06
Honduras	1.03	0.31	1.00	0.35	0.02	1.15	0.23	0.50
India	1.41	1.23	1.25	1.10	0.60	2.43	0.69	0.76
Indonesia	4.28	0.53	9.57	0.52	0.50	12.11	0.37	0.70
Iran (Islamic Republic of)	0.34	0.27	0.30	0.21	0.17	0.45	0.18	0.54
Iraq	2.09	1.48	1.82	0.85	0.94	9.08	1.00	0.17
Jamaica	0.03	0.02	0.04	0.02	0.03	0.05	0.10	0.32
Jordan	0.29	0.14	0.27	0.13	0.25	0.81	0.13	0.29
Kazakhstan	0.08	0.16	0.08	0.09	0.48	0.58	0.37	0.10
Kenya	0.23	0.04	0.11	0.08	0.44	1.24	0.43	0.20
Kiribati	0.77	0.13	0.83	0.26	0.43	2.40	0.14	0.51
Kyrgyzstan	0.01	0.02	0.01	0.01	0.38	0.40	0.16	-0.12
Lao People's Democratic Republic	10.95	1.93	13.16	3.60	3.96	36.58	0.84	2.93
Lebanon	0.37	0.14	0.39	0.25	0.18	0.65	0.20	0.42
Lesotho	0.25	0.03	0.14	0.06	0.81	1.21	0.32	0.26
Liberia	2.31	0.61	2.43	0.63	0.19	5.52	0.36	0.41
Libya	0.31	0.24	0.26	0.19	0.22	0.41	0.14	0.54
Madagascar	0.82	0.84	1.66	0.37	1.06	3.05	0.69	0.26
Malawi	1.21	0.08	0.94	0.17	0.30	1.41	2.10	0.06
Malaysia	0.15	0.01	0.04	0.02	0.06	0.08	0.23	0.26
Maldives	0.66	0.09	0.49	0.19	0.52	0.99	0.16	0.53
Mali	1.70	0.93	2.74	1.10	1.60	8.72	0.07	0.55
Marshall Islands	0.17	0.04	0.14	0.04	0.02	0.20	0.07	0.09
Mauritania	3.41	1.49	4.66	1.59	0.57	11.02	0.32	1.55
Mauritius	-0.04	-0.10	-0.10	-0.10	0.06	-0.07	0.14	0.11
Mexico	0.63	0.19	0.52	0.13	0.06	0.19	0.19	0.27
Micronesia (Federated States of)	0.30	0.07	0.25	0.09	0.08	0.47	0.06	0.11
Mongolia	0.05	0.23	0.05	0.04	0.79	0.85	0.21	0.17
Montenegro	0.11	0.51	0.12	0.34	0.33	0.51	0.07	0.34
Morocco	3.06	1.25	3.92	0.95	1.32	9.43	0.25	2.11
Mozambique	1.70	0.21	1.65	0.26	0.52	3.33	-0.01	0.12
Myanmar	3.46	0.32	7.90	0.50	0.16	11.95	0.72	0.46
Namibia	0.19	0.09	0.18	0.18	0.24	0.36	0.67	0.40
Nepal	14.95	8.01	15.96	5.46	6.12	46.31	1.58	2.91
Nicaragua	0.64	0.25	0.70	0.25	0.18	0.66	0.19	0.24
Niger	3.66	1.68	4.84	1.50	5.52	10.58	-0.19	2.10
Nigeria	1.03	0.48	1.01	0.49	0.45	1.76	0.62	0.17
North Macedonia	0.12	0.09	0.10	0.08	0.42	0.44	0.15	0.42
Pakistan	5.74	3.71	7.07	2.97	1.30	21.32	0.42	1.58
Palestine	0.16	0.10	0.38	0.10	0.05	0.17	0.54	0.70
Papua New Guinea	0.73	0.12	0.32	0.20	0.10	0.90	0.25	0.07
Paraguay	0.32	0.12	0.50	0.20	0.31	0.60	0.20	0.56

	Urine sample	Weight measured	Blood sample	Blood pressure measured	Iron supplementation	ANC content proportion	Early ANC initiation	Mean ANC visits
Peru	2.31	0.48	2.68	0.55	1.63	8.61	0.33	1.32
Philippines	1.13	0.14	1.64	0.10	0.19	2.60	0.45	0.19
Republic of Moldova	0.02	0.07	0.02	0.02	0.10	0.14	0.22	0.12
Rwanda	25.15	0.21	9.55	0.66	2.90	80.05	11.54	0.73
Saint Lucia	0.01	0.02	0.21	0.01	0.07	0.17	0.15	0.29
Saint Vincent and the Grenadines	0.06	0.06	0.06	0.05	0.03	0.08	0.12	0.29
Samoa	0.22	0.04	0.30	0.09	0.24	0.55	0.15	0.46
Sao Tome and Principe	0.18	0.03	0.22	0.03	0.11	0.24	0.47	0.62
Senegal	0.85	0.28	2.48	0.24	0.19	3.69	0.38	0.96
Serbia	0.07	0.08	0.04	0.06	0.37	0.37	0.17	0.25
Sierra Leone	2.89	0.66	2.84	0.76	0.28	5.10	0.57	0.42
Solomon Islands	0.63	0.23	0.72	0.32	0.22	1.38	0.10	0.43
Somalia	3.09	0.96	1.98	1.26	0.85	7.32	0.36	0.04
South Africa	0.01	0.01	0.01	0.01	0.04	0.03	0.99	0.10
South Sudan	1.56	0.50	2.71	1.18	0.91	7.59	0.25	0.97
Sri Lanka	0.03	0.02	0.26	0.02	0.02	0.22	0.20	0.12
Sudan	3.17	2.02	3.67	2.63	0.75	12.37	0.47	1.52
Suriname	0.05	0.00	0.07	-0.01	0.29	0.28	0.20	0.42
Syrian Arab Republic	4.39	1.41	4.17	0.99	0.61	13.84	0.26	0.58
Tajikistan	0.35	0.39	0.30	0.23	0.96	1.19	0.27	0.51
Thailand	0.07	0.07	0.08	0.07	0.13	0.20	0.23	0.42
Timor-Leste	8.31	0.52	9.52	0.50	1.66	9.58	0.84	0.74
Togo	0.39	0.04	0.97	0.08	0.17	1.89	0.67	0.27
Tonga	0.10	0.03	0.64	0.25	0.02	1.14	0.03	0.13
Tunisia	1.35	0.30	0.68	0.24	0.71	1.75	0.23	0.80
Turkmenistan	0.03	0.12	0.02	0.02	0.14	0.15	0.24	0.32
Tuvalu	0.97	0.16	1.09	0.33	0.40	3.16	0.36	0.69
Türkiye	1.29	1.50	1.18	0.60	0.60	2.36	0.41	1.48
Uganda	4.39	0.36	4.00	0.54	0.57	8.01	1.07	0.14
Ukraine	0.08	0.08	0.08	0.08	0.36	0.45	0.12	0.44
United Republic of Tanzania	0.64	-0.05	0.37	-0.01	0.49	1.41	2.56	-0.10
Uzbekistan	0.07	0.17	0.07	0.07	0.40	0.45	0.12	0.27
Vanuatu	0.50	0.22	0.56	0.31	0.29	1.03	0.12	0.51
Venezuela (Bolivarian Republic of)	0.18	0.02	0.19	0.02	0.26	0.33	0.08	0.15
Viet Nam	6.37	1.04	12.87	2.40	0.96	23.06	0.47	3.22
Yemen	2.83	2.96	2.41	1.72	1.71	8.42	0.27	0.91
Zambia	2.64	0.11	1.08	0.20	0.29	3.50	2.99	0.07
Zimbabwe	-0.15	-0.03	0.16	0.03	0.26	0.14	0.40	-0.05

Receipt of all interventions versus early initiation

Supplementary Figure 21. Receipt of all interventions versus early initiation

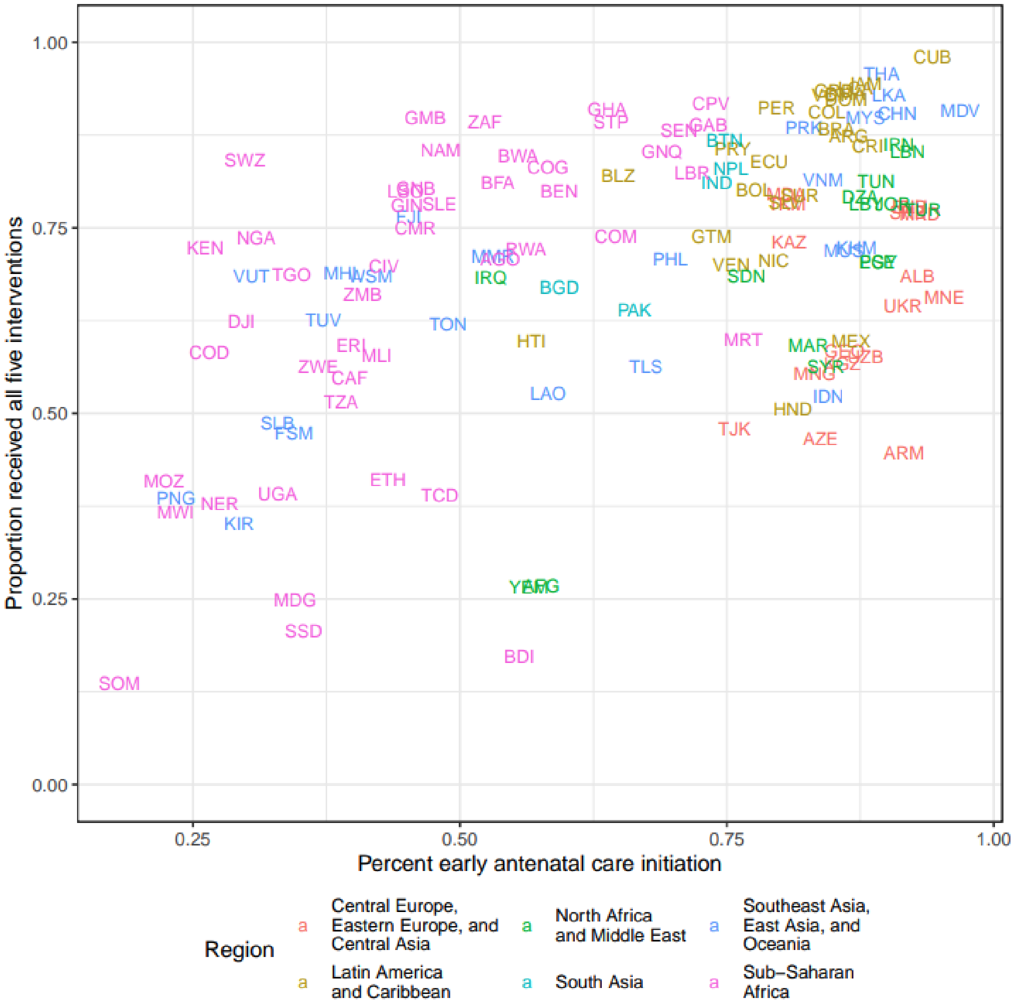

Difference between proportion that attended any ANC and ANC content proportion among women with a live birth, 2023

Supplementary Figure 22. Difference between proportion that attended any ANC and ANC content proportion, 2023

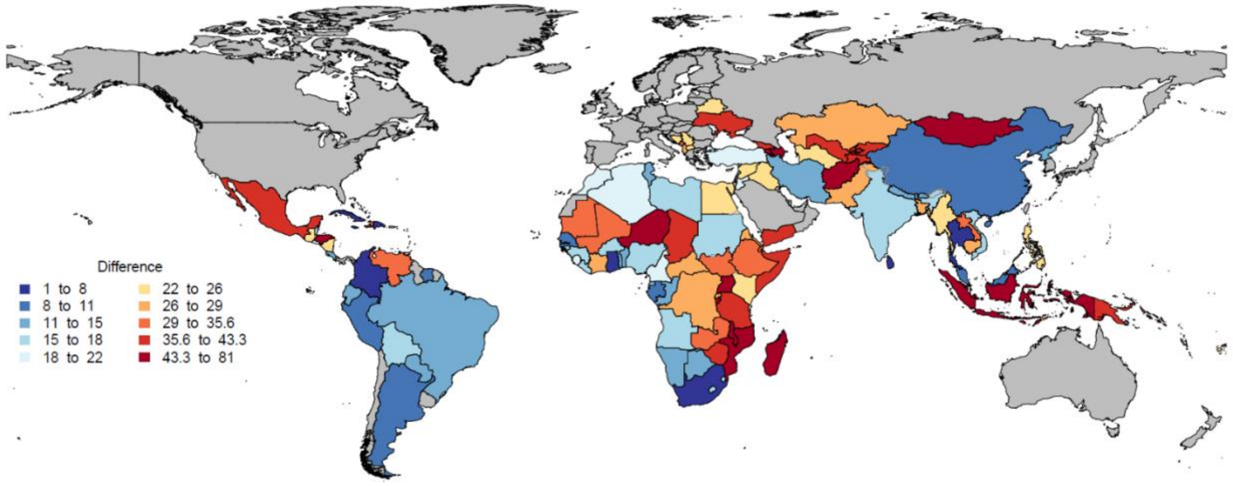

Antenatal content and timing and health outcomes analysis

Supplementary Table 11. Regressions of antenatal content and timing on health outcomes

	Dependent variable:				
	Neonatal mortality rate			Maternal mortality ratio	
	1	2	3	4	5
HAQ	-0.302*** (0.015)	-0.279*** (0.014)	-0.238*** (0.014)	-0.350*** (0.025)	-0.238*** (0.024)
Fac delivery	-0.351*** (0.015)	-0.198*** (0.017)	-0.253*** (0.017)		
SBA				-0.338*** (0.022)	-0.126*** (0.025)
% births over 35	0.066*** (0.010)	0.054*** (0.009)	0.034*** (0.009)	0.228*** (0.015)	0.191*** (0.015)
Adolescent ASFR	0.205*** (0.012)	0.199*** (0.012)	0.191*** (0.011)		
HIV mort				0.113*** (0.014)	0.122*** (0.013)
Hosp. beds				-0.061*** (0.015)	-0.077*** (0.014)
ANC4 coverage	-0.155*** (0.014)			-0.015 (0.024)	
ANC content mean		-0.336*** (0.017)	-0.022 (0.028)		-0.343*** (0.027)
Blood pressure			-0.117*** (0.028)		
Iron Supp.			-0.042*** (0.016)		
Weight			-0.166*** (0.029)		
Constant	0.000 (0.008)	0.000 (0.007)	0.000 (0.007)	0.000 (0.013)	0.000 (0.012)
Observations	3,799	3,799	3,799	3,799	3,799
R^2	0.779	0.795	0.805	0.397	0.422
Adjusted R^2	0.779	0.794	0.804	0.396	0.421

	Dependent Variable:		
	Stillbirths per 1000 live births		
	7	8	9
Maternal Edu.	-0.066* (0.035)	-0.090** (0.035)	-0.106*** (0.035)
SDI	-0.540*** (0.046)	-0.474*** (0.046)	-0.414*** (0.046)
SBA	-0.309*** (0.019)	-0.211*** (0.022)	-0.195*** (0.019)
Hosp. beds	-0.096*** (0.013)	-0.100*** (0.013)	-0.088*** (0.013)
Adolescent ASFR	-0.132*** (0.022)	-0.118*** (0.021)	-0.108*** (0.021)
ANC4 coverage	-0.040** (0.020)		
ANC mean composite		-0.177*** (0.023)	
Blood pressure			-0.245*** (0.018)
Constant	0.000 (0.011)	0.000 (0.011)	0.000 (0.010)
Observations	3,799	3,799	3,799
R^2	0.571	0.577	0.590
Adjusted R^2	0.570	0.577	0.589

Notes: Standard errors in parentheses. *p<0.1; **p<0.05; ***p<0.01, HAQ: Healthcare access and quality index, Fac delivery: facility delivery rate, SBA: skilled birth attendance rate, Adolescent ASFR: Age-specific fertility rate in adolescents ages 10-19, HIV mort: HIV mortality for females, 10-54, ANC4 coverage: Coverage of at least four ANC visits, ANC content mean: Composite indicator described in study, Blood pressure: blood pressure measured during ANC, Weight: weight measured during ANC

Supplementary Table 12. Relative proportion of variance in health outcomes explained

	Dependent variable:				
	Neonatal mortality rate			Maternal mortality ratio	
	1	2	3	4	5
HAQ	0.2	0.19	0.13	0.09	0.08
	(0.18, 0.20)	(0.18, 0.20)	(0.12, 0.14)	(0.09, 0.11)	(0.08, 0.09)
Facility delivery	0.23	0.21	0.14		
	(0.22, 0.24)	(0.20, 0.22)	(0.14, 0.15)		
SBA				0.15	0.13
				(0.13, 0.18)	(0.11, 0.15)
% births over 35	0.01	0.01	0.01	0.03	0.02
	(0.01, 0.02)	(0.01, 0.02)	(0.01, 0.01)	(0.02, 0.03)	(0.02, 0.03)
Adolescent ASFR	0.17	0.16	0.12		
	(0.16, 0.18)	(0.15, 0.17)	(0.11, 0.13)		
HIV mort				0.03	0.03
				(0.02, 0.05)	(0.02, 0.04)
Hosp. beds				0.01	0.01
				(0.01, 0.01)	(0.01, 0.01)

ANC4 coverage	0.18			0.08	
		(0.17, 0.188)		(0.07, 0.09)	
ANC content mean		0.23	0.14		0.15
		(0.22, 0.23)	(0.13, 0.15)		(0.14, 0.17)
Blood pressure			0.13		
			(0.13, 0.14)		
Weight measured			0.13		
			(0.12, 0.13)		

	Stillbirth rate		
	6	7	8
Maternal Edu.	0.12	0.12	0.12
	(0.11,0.13)	(0.11,0.13)	(0.11,0.13)
SDI	0.14	0.13	0.13
	(0.13,0.15)	(0.13,0.14)	(0.13,0.14)
SBA	0.13	0.12	0.12
	(0.12,0.14)	(0.11,0.13)	(0.11,0.13)
Hosp. beds	0.01	0.01	0.01
	(0.01,0.02)	(0.01,0.01)	(0.01,0.01)
Adolescent ASFR	0.08	0.08	0.08
	(0.07,0.08)	(0.07,0.08)	(0.07,0.08)
ANC4 coverage	0.09		
	(0.09,0.1)		
ANC content mean		0.12	
		(0.11,0.13)	
Blood pressure			0.13
			(0.12,0.14)

Notes: 95% confidence intervals in parentheses. HAQ: Healthcare access and quality index, Fac delivery: facility delivery rate, SBA: skilled birth attendance rate, Adolescent ASFR: Age-specific fertility rate in adolescents ages 10-19, HIV mort: HIV mortality for females, 10-54, SDI: Socio-demographic index, Maternal Edu: maternal education, ANC4 coverage: Coverage of at least four ANC visits, ANC content mean: Composite indicator described in study, Blood pressure: blood pressure measured during ANC, Weight: weight measured during ANC

Antenatal content and timing among women who attended any ANC by country, 2023

Supplementary Figure 23. Indicator coverage among women who attended any ANC by country, 2023

	Urine sample	Weight measured	Blood sample	Blood pressure measured	Iron supplementation	ANC content proportion	Early ANC initiation
All study countries	87	94	91	95	90	74	71
Central Europe, Eastern Europe, and Central Asia	99	97	100	100	65	63	89
Albania	99	93	99	97	77	72	98
Armenia	100	99	100	100	46	45	92
Azerbaijan	97	88	98	98	57	51	93
Belarus	100	100	100	100	79	78	93
Bosnia and Herzegovina	99	98	100	99	78	78	93
Georgia	100	100	100	100	60	59	87
Kazakhstan	100	100	100	100	78	73	81
Kyrgyzstan	100	100	100	100	58	57	87
Mongolia	100	96	100	100	56	56	84
Montenegro	100	88	100	99	80	68	98
North Macedonia	99	99	100	99	78	77	94
Republic of Moldova	100	99	100	100	83	80	81
Serbia	99	96	100	99	77	77	92
Tajikistan	98	92	99	99	58	53	84
Turkmenistan	100	96	100	100	85	78	81
Ukraine	100	100	100	100	67	65	92
Uzbekistan	100	96	100	100	59	58	89
Latin America and Caribbean	97	99	97	99	84	81	84
Argentina	100	100	100	100	99	91	90
Belize	100	99	100	100	87	84	67
Bolivia (Plurinational State of)	92	99	92	99	96	85	82
Brazil	99	100	99	100	91	89	86
Colombia	99	100	99	100	96	93	86
Costa Rica	98	100	100	99	88	87	89
Cuba	100	100	100	100	100	99	95
Dominica	100	100	100	100	96	94	87
Dominican Republic	99	100	100	100	96	93	87
Ecuador	96	100	96	100	94	87	82
El Salvador	98	99	99	100	84	81	83
Grenada	100	100	100	100	96	94	86
Guatemala	86	96	85	96	78	76	75
Haiti	77	92	75	93	89	66	70
Honduras	97	99	97	98	56	53	85
Jamaica	100	100	100	100	96	95	89
Mexico	97	99	97	99	61	61	88
Nicaragua	97	100	97	100	78	74	84
Paraguay	98	100	99	99	91	88	78
Peru	98	100	98	100	96	92	81
Saint Lucia	100	100	99	100	96	94	88
Saint Vincent and the Grenadines	100	100	100	100	96	94	85
Suriname	99	100	99	100	100	89	92
Venezuela (Bolivarian Republic of)	90	98	87	97	70	70	76
North Africa and Middle East	89	89	90	96	87	69	89
Afghanistan	64	63	57	90	84	34	73
Algeria	88	97	96	97	88	82	90
Egypt	95	96	96	98	88	76	97
Iran (Islamic Republic of)	99	99	100	100	94	87	93
Iraq	93	87	93	95	90	75	65
Jordan	98	99	99	99	84	80	93
Lebanon	100	100	100	99	95	88	95
Libya	98	97	99	100	94	83	94
Morocco	87	93	93	99	100	76	100
Palestine	98	98	99	99	86	71	90
Sudan	95	88	95	95	100	81	91
Syrian Arab Republic	85	89	86	96	87	69	100
Tunisia	93	98	98	99	93	85	94
Türkiye	97	95	99	99	90	79	95
Yemen	86	77	89	91	55	38	81
South Asia	92	89	92	94	90	80	75
Bangladesh	86	92	85	97	86	70	62
Bhutan	98	96	99	99	97	88	76
India	95	93	95	94	94	84	78
Nepal	92	94	89	99	99	86	78
Pakistan	82	72	86	94	78	69	72
Cambodia	79	98	90	99	99	73	88
China	100	100	100	100	99	92	93
Democratic People's Republic of Korea	98	100	98	100	96	89	83

	Urine sample	Weight measured	Blood sample	Blood pressure measured	Iron supplementation	ANC content proportion	Early ANC initiation
Southeast Asia, East Asia, and Oceania	87	99	89	99	96	80	86
Fiji	94	98	96	98	93	77	46
Indonesia	59	98	70	99	92	54	87
Kiribati	66	91	68	85	84	38	31
Lao People's Democratic Republic	68	97	72	90	100	63	69
Malaysia	99	100	100	100	96	90	88
Maldives	100	100	100	100	93	91	97
Marshall Islands	88	96	91	96	95	71	40
Mauritius	98	100	100	100	100	90	100
Micronesia (Federated States of)	81	95	80	91	100	58	43
Myanmar	85	98	86	97	100	77	58
Papua New Guinea	58	93	85	91	98	50	31
Philippines	86	99	85	100	91	76	75
Samoa	92	98	90	95	93	73	44
Solomon Islands	78	95	74	89	95	52	35
Sri Lanka	100	100	97	100	99	94	91
Thailand	100	100	100	100	97	97	90
Timor-Leste	73	100	72	99	100	66	80
Tonga	95	98	73	85	95	63	50
Tuvalu	86	96	86	93	91	66	39
Vanuatu	91	97	92	96	96	77	34
Viet Nam	91	97	88	94	98	83	86
Sub-Saharan Africa	81	96	89	92	89	66	44
Angola	91	98	99	93	96	81	62
Benin	96	99	94	99	100	88	65
Botswana	99	100	100	100	95	86	57
Burkina Faso	90	100	94	95	98	83	55
Burundi	39	98	89	66	56	18	57
Cabo Verde	100	100	100	100	98	92	74
Cameroon	95	99	98	98	87	81	49
Central African Republic	90	95	90	92	82	66	49
Chad	57	92	66	87	75	48	60
Comoros	94	98	95	95	93	77	67
Congo	98	99	100	99	91	87	61
Côte d'Ivoire	90	98	90	97	94	73	45
Democratic Republic of the Congo	80	95	87	90	80	68	31
Djibouti	95	97	96	98	67	64	31
Equatorial Guinea	99	100	100	100	96	89	72
Eritrea	81	96	86	96	84	69	47
Eswatini	96	100	100	99	92	85	30
Ethiopia	87	95	90	93	78	55	59
Gabon	98	99	99	99	96	91	75
Gambia	96	99	99	99	98	90	47
Ghana	99	99	99	100	94	92	65
Guinea	92	95	91	97	95	82	47
Guinea-Bissau	91	96	94	97	95	83	47
Kenya	86	97	92	94	86	74	28
Lesotho	97	98	98	99	89	84	47
Liberia	93	89	96	98	99	84	73
Madagascar	39	92	51	85	83	28	39
Malawi	44	98	96	91	91	37	24
Mali	77	98	80	96	88	67	49
Mauritania	92	93	85	94	98	67	87
Mozambique	64	97	89	71	91	48	26
Namibia	98	99	99	99	94	88	50
Niger	47	98	58	80	90	41	30
Nigeria	91	95	91	95	92	80	36
Rwanda	91	99	98	95	86	73	57
Sao Tome and Principe	98	100	98	100	97	90	65
Senegal	98	99	95	100	99	90	72
Sierra Leone	88	95	96	98	98	79	49
Somalia	74	83	77	94	95	25	35
South Africa	100	99	100	100	97	93	55
South Sudan	49	90	60	58	100	39	69
Togo	94	99	93	98	100	83	42
Uganda	54	92	95	81	92	40	34
United Republic of Tanzania	73	97	93	83	92	58	43
Zambia	78	98	97	97	99	66	41
Zimbabwe	71	94	97	96	86	61	40

References

1 Stevens GA, Alkema L, Black RE, *et al.* Guidelines for accurate and transparent health estimates reporting: the GATHER statement. *The Lancet* 2016; **388**: e19–23.

2 Zheng P, Barber ,Ryan, Sorensen ,Reed J. D., Murray ,Christopher J. L., and Aravkin AY. Trimmed Constrained Mixed Effects Models: Formulations and Algorithms. *Journal of Computational and Graphical Statistics* 2021; **30**: 544–56.

3 Hay SI, Ong LK, Santomauro DF, *et al.* Global burden of 375 diseases and injuries, disability adjusted life years, risk-attributable burden of 88 risk factors, and healthy life expectancy in 204 countries and territories, including 660 subnational locations, 1990-2023: a systematic analysis for the Global Burden of Disease Study 2023. *The Lancet* https://www.thelancet.com/journals/lancet/article/PIIS0140-6736(24)00933-4/fulltext (accessed May 13, 2025).

4 Murray CJ, Ezzati M, Flaxman AD, *et al.* GBD 2010: design, definitions, and metrics. *The Lancet* 2012; **380**: 2063–6.

5 Haakenstad A, Yearwood JA, Fullman N, *et al.* Assessing performance of the Healthcare Access and Quality Index, overall and by select age groups, for 204 countries and territories, 1990–2019: a systematic analysis from the Global Burden of Disease Study 2019. *The Lancet global health* 2022; **10**: e1715–43.

6 Institute For Health Metrics And Evaluation. Global Burden of Disease Study 2021 (GBD 2021) Covariates 1980-2021. 2024. DOI:10.6069/B09T-1R53.

7 Haakenstad A, Irvine CMS, Knight M, *et al.* Measuring the availability of human resources for health and its relationship to universal health coverage for 204 countries and territories from 1990 to 2019: a systematic analysis for the Global Burden of Disease Study 2019. *The Lancet* 2022; **399**: 2129–54.

8 Naghavi M, Ong KL, Aali A, *et al.* Global burden of 288 causes of death and life expectancy decomposition in 204 countries and territories and 811 subnational locations, 1990–2021: a systematic analysis for the Global Burden of Disease Study 2021. *The Lancet* 2024; **403**: 2100–32.

9 Schumacher AE, Kyu HH, Aali A, *et al.* Global age-sex-specific mortality, life expectancy, and population estimates in 204 countries and territories and 811 subnational locations, 1950–2021, and the impact of the COVID-19 pandemic: a comprehensive demographic analysis for the Global Burden of Disease Study 2021. *The Lancet* 2024; **403**: 1989–2056.

10 Groemping U. Relative Importance for Linear Regression in R: The Package relaimpo. *Journal of Statistical Software* 2007; **17**: 1–27.

11 Bhattacharjee NV, Schumacher AE, Aali A, *et al.* Global fertility in 204 countries and territories, 1950–2021, with forecasts to 2100: a comprehensive demographic analysis for the Global Burden of Disease Study 2021. *The Lancet* 2024; **403**: 2057–99.

12 Friedman J, York H, Graetz N, *et al.* Measuring and forecasting progress towards the education-related SDG targets. *Nature* 2020; **580**: 636–9.
